# Supplementary material for: Study protocol for the epigenetic characterization of angor pectoris according to the affected coronary compartment: Global and comprehensive assessment of the relationship between invasive coronary physiology and microRNAs
Source: PLoS One. 2023 May 11;18(5):e0283097. doi: 10.1371/journal.pone.0283097 (PMC10174526; doi:10.1371/journal.pone.0283097)
Supplement: S1 Checklist — (DOC) [file pone.0283097.s001.doc]

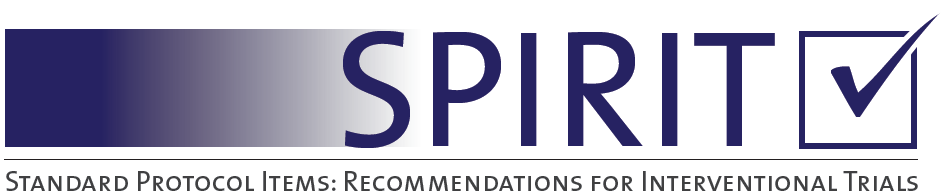


SPIRIT 2013 Checklist: Recommended items to address in a clinical trial protocol and related documents*

| Section/item | ItemNo | Description |
| --- | --- | --- |
| **Administrative information** | | |
| Title | 1 | Study protocol for the epigenetic characterization of angor pectoris according to the affected coronary compartment: global and comprehensive assessment of the relationship between invasive physiological coronary assessment and microRNAs. |
| Trial registration | 2a | ClinicalTrials.gov (NCT number: NCT05374694). |
| Protocol version | 3 | March 31st, 2022. Version 1 |
| Funding | 4 | DdGC (Miguel Servet 2020: CP20/00041) has received financial support from Instituto de Salud Carlos III co-funded by the European Social Fund (ESF)/ “Investing in your future”. |
| Roles and responsibilities | 5a | Lucía Matute-Blanco. Department of Cardiology, University Hospital Arnau de Vilanova, Institut de Reçerca Biomèdica de Lleida (IRBLleida), Lleida, Spain. CR: Conceptualization, Data curation, Formal analysis, Investigation, Methodology, Project administration, Resources, Software, Supervision, Validation, Visualization, Writing – original draft, Writing – review & editing.  Diego Fernández-Rodríguez. Department of Cardiology, University Hospital Arnau de Vilanova, Institut de Reçerca Biomèdica de Lleida (IRBLleida), Lleida, Spain. CR: Conceptualization, Data curation, Formal analysis, Investigation, Methodology, Project administration, Resources, Software, Supervision, Validation, Visualization, Writing – original draft, Writing – review & editing.  Juan Casanova-Sandoval. Department of Cardiology, University Hospital Arnau de Vilanova, Institut de Reçerca Biomèdica de Lleida (IRBLleida), Lleida, Spain. CR: Investigation, Writing – review & editing.  Thalía Belmonte. TRMM Group, Institut de Reçerca Biomèdica de Lleida (IRBLleida), Lleida, Spain.CIBER of Respiratory Diseases (CIBERES), Institute of Health Carlos III, Madrid, Spain. CR: Investigation, Writing – review & editing.  Kristian Rivera. Department of Cardiology, University Hospital Arnau de Vilanova, Institut de Reçerca Biomèdica de Lleida (IRBLleida), Lleida, Spain. CR: Investigation, Writing – review & editing.  Marcos Garcia-Guimaraes. Department of Cardiology, University Hospital Arnau de Vilanova, Institut de Reçerca Biomèdica de Lleida (IRBLleida), Lleida, Spain.CR: Investigation, Writing – review & editing.  Carlos Cortés Villar. Cardiology, University Hospital Miguel Servet, Zaragoza, Spain. CR: Investigation, Writing – review & editing.  Vicente Peral Disdier. Cardiology, University Hospital Son Espases, Palma de Mallorca, Spain.CR: Investigation, Writing – review & editing.  Raúl Millán Segovia. Cardiology, University Hospital Son Espases, Palma de Mallorca, Spain. CR: Investigation, Writing – review & editing.  Ignacio Barriuso. Department of Cardiology, University Hospital Arnau de Vilanova, Institut de Reçerca Biomèdica de Lleida (IRBLleida), Lleida, Spain. CR: Investigation, Writing – review & editing.  David de Gonzalo-Calvo. TRMM Group, Institut de Reçerca Biomèdica de Lleida (IRBLleida), Lleida, Spain. CIBERES, Institute of Health Carlos III, Madrid, Spain. CR: Investigation, Writing – review & editing.  Ferrán Barbè. TRMM Group, Institut de Reçerca Biomèdica de Lleida (IRBLleida), Lleida, Spain. CIBERES, Institute of Health Carlos III, Madrid, Spain. CR: Investigation, Writing – review & editing.  Fernando Worner. Department of Cardiology, University Hospital Arnau de Vilanova, Institut de Reçerca Biomèdica de Lleida (IRBLleida), Lleida, Spain. CR: Investigation, Writing – review & editing. |
|  | 5b | Name and contact information for the trial sponsor – **Not applicable** |
|  | 5c | Role of study sponsor and funders, if any, in study design; collection, management, analysis, and interpretation of data; writing of the report; and the decision to submit the report for publication, including whether they will have ultimate authority over any of these activities – **Not applicable** |
|  | 5d | Composition, roles, and responsibilities of the coordinating centre, steering committee, endpoint adjudication committee, data management team, and other individuals or groups overseeing the trial, if applicable (see Item 21a for data monitoring committee) – **Not applicable** |
| Introduction |  |  |
| Background and rationale | 6a | MicroRNAs are noncoding RNAs involved in post-transcriptional genetic regulation with a proposed role in intercellular communication and are considered promising biomarkers in ischemic heart disease. Invasive physiological evaluation allows a precise assessment of each affected coronary compartment. Although some studies have associated the expression of circulating miRNAs with invasive physiological indexes, their global relationship with coronary compartments has not been evaluated. Here, we will evaluate circulating miRNAs profiles according to the coronary pattern of the vascular compartment affectation. |
|  | 6b | Explanation for choice of comparators – **Not applicable** |
| Objectives | 7 | Hypothesis: Different patterns of coronary artery involvement are associated with the overexpression or underexpression of specific miRNAs.  Primary objective: Characterization of miRNA expression as a function of the coronary compartment in patients with chest pain.  Secondary objectives  1.- To evaluate the association of miRNA expression with the degree of involvement of the stenosis and the extent of epicardial coronary artery.  2.- To evaluate the association of miRNAs levels with angiography-based indices for determination of coronary compartments.  3.- To evaluate the association of miRNA expression with the presence of cardiovascular risk factors and/or established vascular disease.  4.- To evaluate the association of miRNA expression with other hematological and biochemical markers. |
| Trial design | 8 | Multicenter descriptive study. |
| Methods: Participants, interventions, and outcomes | | |
| Study setting | 9 | Multicenter descriptive study to be conducted in three university hospitals in Spain. S1 File |
| Eligibility criteria | 10 | Inclusion criteria:   1. Age ≥ 18 years. 2. Patients with chest pain suggestive of angina evaluated by a cardiologist referred for diagnostic coronary angiography and eventual coronary angioplasty. 3. Echocardiogram ruling out non-coronary cardiac causes of chest pain. 4. Informed consent.   Exclusion criteria:   1. Contrary to allergy not susceptible to receive pre-medication. 2. Severe bronchial asthma or intolerance to adenosine. 3. Atrioventricular block (≥ 2nd degree) or intolerance to acetylcholine. 4. Acute myocardial infarction with elevation of the ST segment. 5. Acute myocardial infarction without elevation of the ST segment. 6. Cardiogenic shock. 7. Total occlusion of any coronary artery that prevents measurement with pressure-temperature guides. 8. Previous coronary bypass. 9. Women who may be pregnant. 10. Renal dysfunction with estimated glomerular filtration rate < 30 mL/min/1.73m2. 11. Inability to understand the nature of the study and/or sign the informed consent. 12. Any other medical condition that, in the opinion of the investigator, may cause safety problems for the patients or may alter the results of the study. |
| Interventions | 11a | Interventions for each group with sufficient detail to allow replication, including how and when they will be administered – **Not applicable** |
|  | 11b | Criteria for discontinuing or modifying allocated interventions for a given trial participant (eg, drug dose change in response to harms, participant request, or improving/worsening disease) – **Not applicable** |
|  | 11c | Strategies to improve adherence to intervention protocols, and any procedures for monitoring adherence (eg, drug tablet return, laboratory tests) – **Not applicable** |
|  | 11d | Relevant concomitant care and interventions that are permitted or prohibited during the trial – **Not applicable** |
| Outcomes | 12 | Primary objective: Characterization of miRNA expression as a function of the coronary compartment in patients with chest pain.  Secondary objectives  1.- To evaluate the association of miRNA expression with the degree of involvement of the stenosis and the extent of epicardial coronary artery.  2.- To evaluate the association of miRNAs levels with angiography-based indices for determination of coronary compartments.  3.- To evaluate the association of miRNA expression with the presence of cardiovascular risk factors and/or established vascular disease.  4.- To evaluate the association of miRNA expression with other hematological and biochemical markers. |
| Participant timeline | 13 | See Figure 2: Timeline and flowchart of the study |
| Sample size | 14 | Because studies evaluating the association between circulating miRNAs and coronary patterns are currently lacking, the sample size was not calculated using standard procedures. Patients of Group 4 (normal coronary indexes) will be used as control group to the Groups 1 to 3 until the recruitment of 25 patients per group will be completed, in a similar amount of patients in similar studies, for a total sample of 100 patients. It should be noted that patients in Group 5 (coronary spasm) may overlap with patients in Groups 3 and 4, so Group 5 has not been considered for sample size estimation |
| Recruitment | 15 | Strategies for achieving adequate participant enrolment to reach target sample size – **Not applicable** |
| **Methods: Assignment of interventions (for controlled trials)** | | |
| Allocation: |  |  |
| Sequence generation | 16a | Method of generating the allocation sequence (eg, computer-generated random numbers), and list of any factors for stratification. To reduce predictability of a random sequence, details of any planned restriction (eg, blocking) should be provided in a separate document that is unavailable to those who enrol participants or assign interventions– **Not applicable** |
| Allocation concealment mechanism | 16b | Mechanism of implementing the allocation sequence (eg, central telephone; sequentially numbered, opaque, sealed envelopes), describing any steps to conceal the sequence until interventions are assigned– **Not applicable** |
| Implementation | 16c | Who will generate the allocation sequence, who will enrol participants, and who will assign participants to interventions– **Not applicable** |
| Blinding (masking) | 17a | Who will be blinded after assignment to interventions (eg, trial participants, care providers, outcome assessors, data analysts), and how– **Not applicable** |
|  | 17b | If blinded, circumstances under which unblinding is permissible, and procedure for revealing a participant’s allocated intervention during the trial– **Not applicable** |
| **Methods: Data collection, management, and analysis** | | |
| Data collection methods | 18a | Plans for assessment and collection of outcome, baseline, and other trial data, including any related processes to promote data quality (eg, duplicate measurements, training of assessors) and a description of study instruments (eg, questionnaires, laboratory tests) along with their reliability and validity, if known. Reference to where data collection forms can be found, if not in the protocol- **Not specified** |
|  | 18b | Plans to promote participant retention and complete follow-up, including list of any outcome data to be collected for participants who discontinue or deviate from intervention protocols – **Not applicable** |
| Data management | 19 | Each center will fill an anonymized and predefined case report form (CRF) developed by the investigators. The selected variables are oriented to the cardiovascular risk factors, comorbidities, clinical findings, ischemia detection tests, medications, analytical parameters, echocardiographic and angiographic findings, and invasive physiological indexes (S3 File). All data collected during the study, after deidentification, will be shared. Data obtained through this study may be provided to qualified researchers with academic interest in cardiovascular diseases. Approval of the request and execution of all applicable agreements are prerequisites for the sharing of data with the requesting party. |
| Statistical methods | 20a | The statistical software package R (www.r-project.org) will be used for statistical analyses. The characteristics of the study population will be summarized through standard descriptive statistics. Differences in variables between study groups and correlations between continuous variables will be analyzed using the appropriate test (parametric or nonparametric). Linear models with empirical Bayes statistics will be used to evaluate differences in omics levels between groups. A propensity score (PS) will be used for adjustment. A heat map of unsupervised hierarchical clustering, principal component analysis (PCA), and volcano plot will be used to determine whether a miRNA profile could differentiate between study groups. Correlations between continuous variables will be estimated using Spearman rank correlation. In addition, we will use a machine learning process based on random forest to construct miRNAs signatures associated with the outcomes. Variable importance plots will be displayed to illustrate the prediction value of each variable. The two-tailed significance level will be set at <0.05. |
|  | 20b | Methods for any additional analyses (eg, subgroup and adjusted analyses) - **Not specified** |
|  | 20c | Definition of analysis population relating to protocol non-adherence (eg, as randomised analysis), and any statistical methods to handle missing data (eg, multiple imputation) – **Not applicable** |
| **Methods: Monitoring** | | |
| Data monitoring | 21a | Composition of data monitoring committee (DMC); summary of its role and reporting structure; statement of whether it is independent from the sponsor and competing interests; and reference to where further details about its charter can be found, if not in the protocol. Alternatively, an explanation of why a DMC is not needed – **Not applicable** |
|  | 21b | Description of any interim analyses and stopping guidelines, including who will have access to these interim results and make the final decision to terminate the trial – **Not applicable** |
| Harms | 22 | Plans for collecting, assessing, reporting, and managing solicited and spontaneously reported adverse events and other unintended effects of trial interventions or trial conduct – **Not applicable** |
| Auditing | 23 | Frequency and procedures for auditing trial conduct, if any, and whether the process will be independent from investigators and the sponsor – **Not applicable** |
| Ethics and dissemination | | |
| Research ethics approval | 24 | Approval by IRB. |
| Protocol amendments | 25 | Plans for communicating important protocol modifications (eg, changes to eligibility criteria, outcomes, analyses) to relevant parties (eg, investigators, REC/IRBs, trial participants, trial registries, journals, regulators) - **Not specified** |
| Consent or assent | 26a | Investigators. |
|  | 26b | Additional consent provisions for collection and use of participant data and biological specimens in ancillary studies, if applicable – **Not applicable** |
| Confidentiality | 27 | Each center will fill an anonymized and predefined case report form (CRF) developed by the investigators. |
| Declaration of interests | 28 | Investigators have no conflicts of interest related with the study. |
| Access to data | 29 | Investigators. Also, all data collected during the study, after deidentification, will be shared. Data obtained through this study may be provided to qualified researchers with academic interest in cardiovascular diseases. Approval of the request and execution of all applicable agreements are prerequisites for the sharing of data with the requesting party. |
| Ancillary and post-trial care | 30 | Provisions, if any, for ancillary and post-trial care, and for compensation to those who suffer harm from trial participation – **Not applicable** |
| Dissemination policy | 31a | Likewise, a dissemination plan aimed at the general public will be established, and actions will be carried out to inform segments of the population of special interest/vulnerability within the scope of the study. The information obtained will be made available to the scientific world and the general public in collaboration with the IRBLleida Communication Department. IRBLleida has taken several steps to disseminate scientific messages in a clear and understandable way. We will use the IRBLleida website (https://www.irblleida.org/es/) that contains information on projects, publications and research groups. To make research more accessible to society, we will use digital communication platforms. Additionally, some of the researchers are members of the European COST CardioRNA program (https://cardiorna.eu/). One of the working groups of this program is focused purely on the communication of the results obtained. |
|  | 31b | Authorship eligibility guidelines and any intended use of professional writers – **Not applicable** |
|  | 31c | Plans, if any, for granting public access to the full protocol, participant-level dataset, and statistical code – **Not applicable** |
| Appendices |  |  |
| Informed consent materials | 32 | Patient information sheet.  Informed consent form. |
| Biological specimens | 33 | Samples will be stored in the IRBLleida Biobank belonging to the National Biobank Platform. The specific analysis of miRNAs will be performed in the TRRM group laboratory of the Institut de Recerca Biomèdica de Lleida. The analysis of the the rest of biochemical and/or hematological parameters will be performed in the laboratories of the University Hospital Arnau de Vilanova of Lleida. If no differences are observed between miRNAs in the different study groups, the samples will be kept in the IRBLleida Biobank for the assessment of other biomarkers in which the TRRM group has experience, such as lncRNAs, circRNAs, proteins, etc. |

*It is strongly recommended that this checklist be read in conjunction with the SPIRIT 2013 Explanation & Elaboration for important clarification on the items. Amendments to the protocol should be tracked and dated. The SPIRIT checklist is copyrighted by the SPIRIT Group under the Creative Commons “[Attribution-NonCommercial-NoDerivs 3.0 Unported](http://www.creativecommons.org/licenses/by-nc-nd/3.0/)” license.
