# Supplementary material for: Study protocol for the epigenetic characterization of angor pectoris according to the affected coronary compartment: Global and comprehensive assessment of the relationship between invasive coronary physiology and microRNAs
Source: PLoS One. 2023 May 11;18(5):e0283097. doi: 10.1371/journal.pone.0283097 (PMC10174526; doi:10.1371/journal.pone.0283097)
Supplement: S1 File — IRBLleida, Institut de Reçerca Biomèdica de Lleida; TRRM Group, Translational Research in Respiratory Medicine Group. (DOCX) [file pone.0283097.s002.docx]

**S1 File.** List of participant centers, departments/groups and investigators.

| **Center** | **Department/Group** | **Investigator** |
| --- | --- | --- |
| University Hospital Arnau de Vilanova / IRBLleida  (Lleida) | Cardiology Department | Lucía Matute-Blanco |
|  |  | Diego Fernández-Rodríguez |
|  |  | Juan Casanova-Sandoval |
|  |  | Kristian Rivera |
|  |  | Marcos Garcia-Guimaraes |
|  |  | Ignacio Barriuso |
|  |  | Fernando Worner |
|  | TRRM Group | David De Gonzalo-Calvo |
|  |  | Thalía Belmonte |
|  |  | Iván D. Benítez |
|  |  | Ferrán Barbè |
| University Hospital Miguel Servet (Zaragoza) | Cardiology Department | Carlos Cortés Villar |
| Son Espases University Hospital (Palma de Mallorca) | Cardiology Department | Vicente Peral Disdier |
|  |  | Raúl Millán Segovia |

IRBLleida, Institut de Reçerca Biomèdica de Lleida; TRRM Group, Translational Research in Respiratory Medicine Group.
