# Supplementary material for: Study protocol for the epigenetic characterization of angor pectoris according to the affected coronary compartment: Global and comprehensive assessment of the relationship between invasive coronary physiology and microRNAs
Source: PLoS One. 2023 May 11;18(5):e0283097. doi: 10.1371/journal.pone.0283097 (PMC10174526; doi:10.1371/journal.pone.0283097)
Supplement: S2 File — RFR, resting full-cycle ratio; FFR, fractional flow reserve; CFR, coronary flow reserve; IMR, index of microvascular resistance; Tmn, mean transit time. (DOCX) [file pone.0283097.s003.docx]

**S2 File.** Recommendations to perform invasive physiological evaluation^[1,11-16]^

| **RFR, FFR, CFR and IMR measurements will be performed as detailed below.** |
| --- |
| **Catheter Selection and Positioning**  • Use guide catheters (at least 5F) without lateral holes.  • Ensure coaxial cannulation of the guiding catheter at the coronary ostium.  • Decannulate the guiding catheter from the ostium to calibrate pressure, equalize and record Pd / Pa if there is any doubt that the catheter may partially obstruct the ostium. |
| **Calibration**  • Before starting measurements, ensure that the aortic pressure is set to zero correctly (1/3 versus 2/3 chest diameter).  • Purge the pressure guide, place it in a horizontal position at the time of connection/calibration and do not move the pressure guide while performing the process.  • Before equalizing pressures, advance the guidewire until the pressure sensor is positioned exactly at the end of the guide catheter.  • Before equalizing the pressures, flush the guiding catheter with saline to remove the viscous contrast agent.  • Before equalizing pressures, remove the introducer and close the hemostatic valve.  • Pressure curves are usually averaged over three to five heartbeats. Therefore, pressure equalization requires some time, and artifacts should not occur during this time. |
| **Pressure-Temperature Guide Positioning**  • For the evaluation of epicardial lesions (RFR and FFR), the pressure sensor should be placed distal to the main vessel to be analyzed.  • RFR is measured first, followed by FFR, CFR and IMR.  • For the determination of CFR and IMR, the pressure sensor should be placed in the distal segment of the vessel corresponding to the lesion to be evaluated.  • In the case of patients referred for coronary angiography who present a positive ischemia test, FFR, CFR and IMR will also be measured, in addition to the arteries of the lesions to be evaluated, in the arteries compatible with the ischemia detected in the ischemia test. Likewise, in the absence of lesions to be evaluated and a previous positive ischemia test to guide the exploration, the FFR, CFR and IMR will be evaluated in the anterior descending artery.  • A second guidewire, in addition to the pressure guidewire, could cause artifacts and should therefore be avoided.  • Detect artifacts: the sensor could interact with the vessel wall, especially in cases of narrow vessel caliber or severe tortuosity.  • Viscous contrast agent in the coronary artery may affect the pd/pa gradient. |
| **Hyperemia**  • Before advancing the pressure wire, administer nitroglycerin ic (usually 200 mcg) to prevent coronary spasm.  • Medications for hyperemia.  - Intravenous adenosine 140 μg/kg/ min.  • In case of measurement results in the borderline area, an increase in the dose of ev adenosine is possible. However, intravenous doses > 180μg /kg/min may reduce coronary perfusion and are therefore not recommended. |
| **Recording and Evaluation**  • Artifacts should be carefully observed and excluded.  RFR and FFR  • For RFR determination, a minimum of 5 consecutive cardiac cycles are required.  • After administration of intravenous adenosine, pressure values may decrease to a minimum before reaching steady state. Wait until stable FFR values are obtained.  • Values measured during atrioventricular block/adenosine-induced bradycardia should be classified as "not evaluable".  • Ectopic beats may falsify the values obtained, so be sure to obtain values during periods of electrically stable time.  CFR and IMR  • For the determination of CFR and IMR, 3 rapid injections of 3 cc of physiological saline will be made at baseline. Subsequently, during the administration of ev adenosine and once the state of maximum hyperemia is reached, 3 rapid injections of 3cc of physiological saline will be performed again.  • Alterations in saline temperature or saline injection rate may cause abnormal Tmn values that are detected by the software. Carefully evaluate the concordance of these values to assess whether any injection should be repeated.  • To determine the CFR, the Tmn information obtained at baseline and under maximum hyperemia is used. To determine the IMR, only the Tmn information obtained at maximum hyperemia is used. |
| **Acetylcholine test will only be performed for patients in Groups 3 and 4 as detailed below.** |
| • Electrocardiographic monitoring of the patient by 12-lead electrocardiogram.  • Cinefluoroscopy images will be obtained in a cranial projection that adequately displays the anterior descending artery.  • Given the expected diffuse nature of the coronary spasm, it will be performed by increasing injections of acetylcholine up to 3 doses of 2, 20 and 100mcg in the left coronary artery.  • Acetylcholine injections will not be performed in the right coronary given the very high risk of severe bradycardia.  • The intracoronary bolus administration time will be performed slowly for 20 seconds.  • The guiding catheter should then be slowly flushed with saline to prevent abrupt injections of the drug remaining in the catheter at the time of cinefluoroscopy image acquisition.  • After each injection, the presence of symptoms suggestive of angina and similar to those that motivated the study will be evaluated, a cinefluoroscopy image will be acquired and a 12-lead electrocardiogram will be performed.  • If significant angiographic spasm or ST elevation/decrease suggestive of microvascular or macrovascular spasm is documented, progression to the next dose will not be continued.  • In case of significant spasm, as well as at the end of the test, 200mcg of nitroglycerin ic will be administered to abolish the effect of acetylcholine. |

RFR, resting full-cycle ratio; FFR, fractional flow reserve; CFR, coronary flow reserve; IMR, index of microvascular resistance; Tmn, mean transit time.
