# Supplementary material for: Study protocol for the epigenetic characterization of angor pectoris according to the affected coronary compartment: Global and comprehensive assessment of the relationship between invasive coronary physiology and microRNAs
Source: PLoS One. 2023 May 11;18(5):e0283097. doi: 10.1371/journal.pone.0283097 (PMC10174526; doi:10.1371/journal.pone.0283097)
Supplement: S3 File — ACE, angiotensin-converting enzyme; ACS, acute coronary syndrome; aGLP-1, agonist glucagon-like peptide 1; Apo B 100, apolipoprotein B 100; ARA II, angiotensin II receptor antagonists; ARNI, angiotensin receptor/neprilysin inhibitor; CABG, coronary artery bypass graft surgery; CFR, coronary flow reserve; CK, creatine kinase; CPAP, continuous positive airway pressure; CRP, C-reactive protein; DVT, deep vein thrombosis; FFR, fractional flow reserve; GFR, glomerular filtration rate; GGT, gamma-glutamyl transferase; GOT/AST, aspartate aminotransferase; GPT/ALT, alanine aminotransferase; HbA1c, glycated haemoglobin; iDPP4, inhibitor of dipeptidyl peptidase 4; IMR, index of microvascular resistance; iSGLT2, inhibitor sodium-glucose cotransporter-2; HDL, high-density lipoprotein; iPCKS9, proprotein convertase subtilisin/kexin type 9 inhibitor; LA, left atrial; LDH, lactate dehydrogenase; LDL, low-density lipoprotein; LV, left ventricle; MV, mitral valve; NYHA, New York Heart Association; NT-proBNP, N-terminal-pro hormone BNP; Pa, aortic pressure; PCI, percutaneous coronary intervention; Pd, distal intracoronary pressure; PE, pulmonary embolism; PT, prothrombin time; PTT, Partial Thromboplastin Time; RA, right atrial; RFR, resting full-cycle ratio; RV, right ventricle; sPAP, systolic pulmonary arterial pressure; SpO2, peripheral oxygen saturation; STEMI, ST-elevation myocardial infarction; TAPSE, tricuspid annular plane systolic excursion; td, telediastolic; Tmn, mean transit time; TSH, thyroid stimulating hormone; V., velocity; WBC, white blood cells. (DOCX) [file pone.0283097.s004.docx]

**S3 File.** Data to be obtained in the study.

| Baseline characteristics | Medications | Analytical parameters | Echocardiographic findings | Physiological parameters |
| --- | --- | --- | --- | --- |
| Risk factors and comorbidities  • Age  • Sex  • Weight  • Height  • Hypertension  • Diabetes mellitus  • Hypercholesterolemia  • Smoking status  • Alcohol consumption  • Atrial fibrillation/ Atrial flutter  • Heart failure  • NYHA class  • Previous valve replacement  • Familial ischemic heart disease  • Previous Stroke  • Previous ACS  • Type ACS  • STEMI localization  • Previous PCI  • Previous CABG  • Peripheral vascular disease  • Chronic obstructive pulmonary disease  • Sleep apnea syndrome  • CPAP  • Pulmonary arterial hypertension  • Previous PE and DVT  • Major bleeding  • Cancer  • Frailty Charlson Comorbidity Index  Clinical findings  • Exercise-induce angina  • Angina at rest  • Date of angina onset  Ischemia detection test  • Test type  • Ischemia induction  • Localization of ischemia induction | • Aspirin  • P2Y12 Inhibitor  • ACE inhibitor  • ACE inhibitor type  • Total daily ACE inhibitor dose  • ARA II  • Type of ARA II  •Total daily ARA II dose  • Mineralocorticoids antagonist  • Type of mineralocorticoids antagonist  • Mineralocorticoids antagonist dose  • ARNI  • Total daily ARNI dose  • Beta-blockers  • Type of beta-blockers  • Beta-blocker dose  • Calcium antagonist  • Type of calcium antagonist  • Calcium antagonist dose  • Nitrates  • Nitrates dose  • Statins  • Type of statins  • Statins dose  • Ezetimibe  • iPCSK9  • Metformin  • iSGLT2  • iDPP4  • aGLP-1  • Insulin  • Diuretics  • Anti-vitamins  • Direct acting oral anticoagulants  • Proton pump inhibitor  • Oral iron  • Antidepressives | • Glucose  • Creatinine  • GFR  • Cholesterol  • LDL  • HDL  • Apo B 100  • Triglycerides  • CK  • Troponin I hs  • GOT/AST  • GPT/ALT  • GGT  • Alkaline phosphatase  • Total bilirubin  • LDH  • Iron  • Ferritin  • Transferrin saturation index  • Transferrin  • CRP  • NT-proBNP  • TSH  • Folate  • Vitamin B12  • WBC  • Hemoglobin  • Hematocrit  • Platelets  • HBA1c  • PTT  • PTT (ratio)  • PT (ratio)  • Fibrinogen | • LV end-diastolic diameter  • LV end-systolic diameter  • LV td septum  • LV posterior wall td  • LV ejection fraction  • LV td mass  • LV mass td index  • Segmentary contractility alterations  • Segmental contractility alteration localization  • V. max E MV  • V. max A MV  • E/A MV  • MV deceleration time  • E/E' average  • LA diameter  • LA index volume  • sPAP  • RV basal diameter  • TAPSE  • S' wave  • RA index. volume  • Aortic root diameter  • Ascending aortic diameter  • Aortic arch diameter  • Mitral insufficiency  • Etiology of mitral insufficiency  • Mitral Stenosis  • Etiology of mitral stenosis  • Aortic regurgitation  • Etiology of aortic regurgitation  • Aortic Stenosis  • Etiology of aortic stenosis  • Tricuspid insufficiency | • Basal heart rate  • Hyperemia heart rate  • SpO2  • Basal Pa  • Hyperemia Pa  • Basal Pd  • Hyperemia Pd  • Basal Pd/Pa  • RFR  • FFR  • CFR  • IMR  • Basal Tmn  • Hyperemia Tmn  • CFR normalized  • IMR corrected  • RRR  • Coronary vasospasm test |
|  |  |  |  |  |
|  |  |  |  |  |
|  |  |  |  | Angiographic findings |
|  |  |  |  | • Coronary dominance  • Angiographic disease  • Vessel examined  • Reference vessel size, (mm)  • Diameter stenosis (%)  • Length stenosis, (mm) |

ACE, angiotensin-converting enzyme; ACS, acute coronary syndrome; aGLP-1, agonist glucagon-like peptide 1; Apo B 100, apolipoprotein B 100; ARA II, angiotensin II receptor antagonists; ARNI, angiotensin receptor/neprilysin inhibitor; CABG, coronary artery bypass graft surgery; CFR, coronary flow reserve; CK, creatine kinase; CPAP, continuous positive airway pressure; CRP, C-reactive protein; DVT, deep vein thrombosis; FFR, fractional flow reserve; GFR, glomerular filtration rate; GGT, gamma-glutamyl transferase; GOT/AST, aspartate aminotransferase; GPT/ALT, alanine aminotransferase; HbA1c, glycated haemoglobin; iDPP4, inhibitor of dipeptidyl peptidase 4; IMR, index of microvascular resistance; iSGLT2, inhibitor sodium-glucose cotransporter-2; HDL, high-density lipoprotein; iPCKS9, proprotein convertase subtilisin/kexin type 9 inhibitor ; LA, left atrial; LDH, lactate dehydrogenase; LDL, low-density lipoprotein; LV, left ventricle; MV, mitral valve; NYHA, New York Heart Association; NT-proBNP, N-terminal-pro hormone BNP; Pa, aortic pressure; PCI, percutaneous coronary intervention; Pd, distal intracoronary pressure; PE, pulmonary embolism; PT, prothrombin time; PTT, Partial Thromboplastin Time; RA, right atrial; RFR, resting full-cycle ratio; RV, right ventricle; sPAP, systolic pulmonary arterial pressure; SpO2, peripheral oxygen saturation; STEMI, ST-elevation myocardial infarction; TAPSE, tricuspid annular plane systolic excursion; td, telediastolic; Tmn, mean transit time; TSH, thyroid stimulating hormone ; V., velocity; WBC, white blood cells.
