# Supplementary material for: Study protocol for the epigenetic characterization of angor pectoris according to the affected coronary compartment: Global and comprehensive assessment of the relationship between invasive coronary physiology and microRNAs
Source: PLoS One. 2023 May 11;18(5):e0283097. doi: 10.1371/journal.pone.0283097 (PMC10174526; doi:10.1371/journal.pone.0283097)
Supplement: S4 File — (DOCX) [file pone.0283097.s005.docx]

**Epigenetic characterization of angina pectoris according to the coronary compartment affected: relationship between invasive physiological coronary assessment and microRNAs.**

**Principal investigator:** Lucía Matute-Blanco^a^.

**Collaborating Researchers:** David De Gonzalo-Calvo^b^, Juan Casanova-Sandoval^a^, Thalía Belmonte García^b^, Diego Fernández-Rodríguez^a^, Kristian Rivera^a^, Ignacio Barriuso^a^, Ferrán Barbé ^a,c^, Fernando Worner Diz ^a,b^.

**Affiliations:**

Cardiology department. University Hospital Arnau de Vilanova, Lleida^a^.

Grup TRRM. Institut de Recerca Biomèdica de Lleida (IRB LLEIDA). University Hospital Arnau de Vilanova, Lleida^b^.

Pneumology department. University Hospital Arnau de Vilanova, Lleida^c^.

**Address:** Avenida Rovira Roure 80. Lleida.

**Zip code:** 25198.

**Phone:** +34-973-468024

**Index**

1. Introduction
2. Background
   1. Invasive physiological assessment of the coronary circulation.
      1. Coronary Flow Reserve (CFR).
      2. Fractional Flow Reserve (FFR).
      3. Index of Microvascular Resistance (IMR).
      4. Fractional Flow Reserve (FFR).
      5. "Resting Full-Cycle Ratio" (RFR) and "Adjusted Resting Full-Cycle Ratio" (RFRa).
      6. Computational angiographic models: Quantitative Flow Ratio (QFR) and Angiography-derived Index of Microcirculatory Resistance (IMRangio).
      7. Coronary vaso-reactivity test with acetylcholine.
   2. MicroRNAs and Cardiovascular Disease
      1. Synthesis and function of microRNAs.
      2. Potential of microRNAs as biomarkers.
      3. Regulation of gene expression in cardiovascular disease: role of microRNAs.
      4. Relationship between microRNAs and cardiovascular disease: summary of evidence.
      5. Variability of microRNAs in published studies
3. Hypothesis
4. Primary objective
5. Secondary objectives
6. Material and methods.
   1. Recruitment.
   2. Inclusion criteria.
   3. Exclusion criteria.
   4. Sample size estimation.
   5. Statistical analysis
   6. Selection of microRNAs and other samples.
   7. Collection, processing, preservation, and analysis of biological samples.
   8. Bioinformatics analysis.
   9. Design.
   10. Diagnostic coronary angiography and physiological evaluation of the coronary tree.
   11. Determination of the affected coronary pattern.

7. Scientific interest of the research.

8. Analysis of the feasibility of the project.

9. References.

10. Annexes.

**INTRODUCTION.**

Chest pain is one of the main reasons for cardiology consultation, reflecting the existence of ischemic heart disease. (1). However, in a high proportion of patients with myocardial ischemia, invasive tests do not document obstructive coronary lesions of the epicardial coronary arteries. (1,2).

In the coronary arterial circulation there is progressive branching of the vessels, constituting two distinct compartments that can be involved individually or in combination: a) the "macrovascular compartment" constituted by the epicardial arteries (conduction function); and b) the "microvascular compartment" constituted by the arterioles (flow regulatory function) and the capillaries (exchange function). Technological progress has made possible the development of new invasive physiological coronary indices that allow an exhaustive examination of both arterial compartments, making it possible to precisely discern their involvement. (2–5).

On the other hand, microRNAs (miRNAs) comprise a range of small fragments of noncoding RNA (nc-RNA) that influence post-transcriptional regulation of gene expression; their expression has been shown to be altered in ischemic heart disease (6). However, there are no research studies that comprehensively evaluate the role of miRNAs in relation to the affected coronary compartment (7).

Thus, our objective will be to prospectively evaluate the relationship of the different patterns of coronary involvement with the levels of circulating miRNAs.

**BACKGROUND**

**- Invasive physiological evaluation of the coronary circulation.**


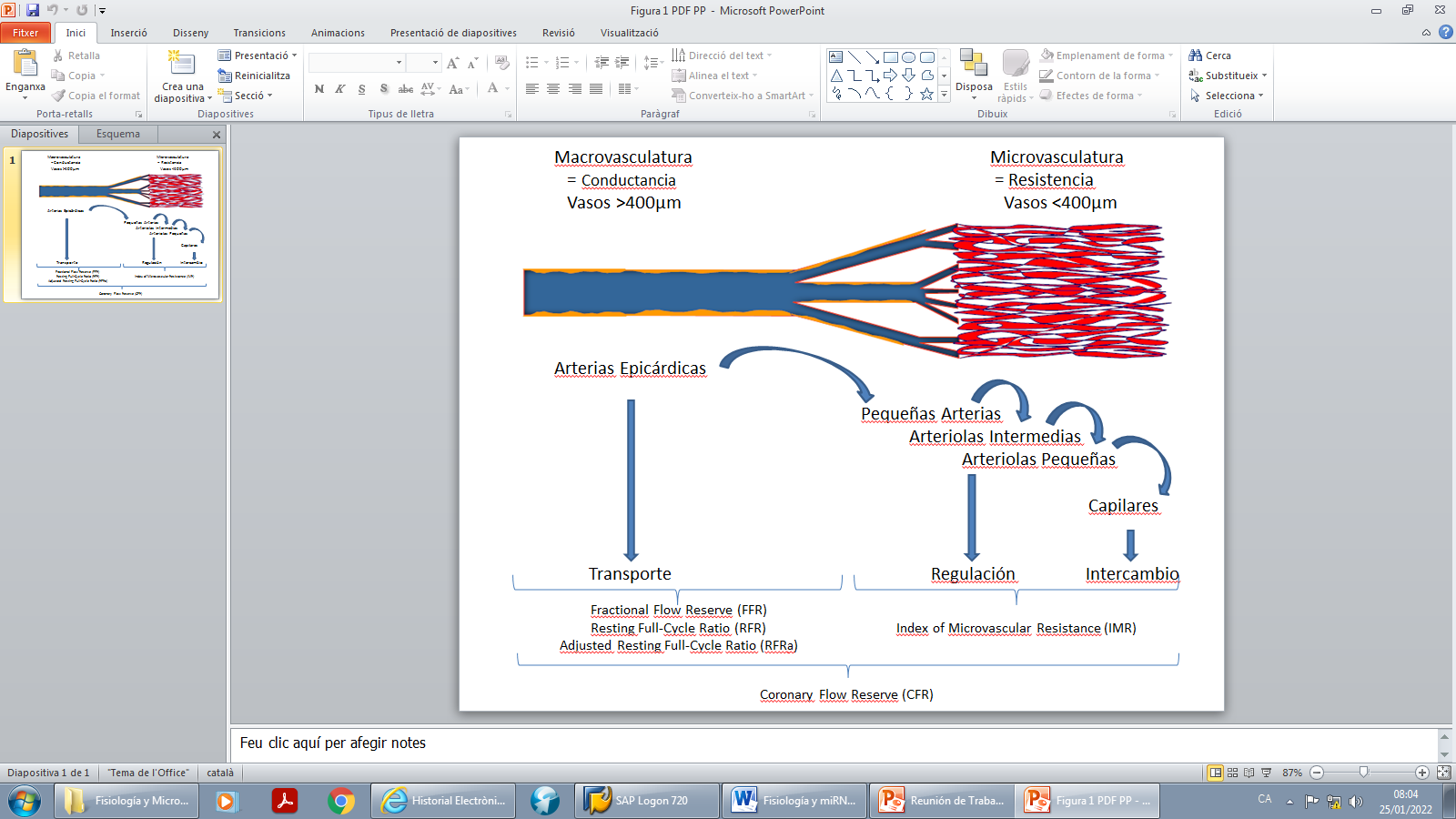
 Coronary circulation is the set of vessels responsible for supplying oxygen- and nutrient-rich blood to the myocardium, and cardiac function being highly dependent on the maintenance and modulation of coronary flow. The myocardium is one of the tissues with the highest basal aerobic requirements in the body (8-10 mL O2/min/100 g in the myocardium vs. 0.15 mL O2/min/100 g in skeletal muscle) (3,8). At the anatomical level, coronary flow regulation is structured in two distinct compartments. On the one hand, there is the macrovascular compartment comprising arteries > 400 microns (conductance vessels) whose function is to distribute coronary flow to different territories, and on the other, the microvascular compartment comprising arteries < 400 microns, intermediate arterioles and small arterioles (resistance vessels) whose function is to regulate the amount of coronary flow, by vasodilation or vasoconstriction, that reaches the capillaries in which the exchange of nutrients and cellular waste takes place. (3,8).

*Illustration 1. Physiology of the coronary circulation.*

Hemodynamic studies on coronary blood circulation are based on "Poiseuille's Law" for hemodynamic circuits and its relation to "Ohm's Law" for electrical circuits. Based on these, it is established that the flow of fluid through a circuit is related proportionally to the pressure gradient across the circuit and inversely proportional to the resistance of the circuit (9).

| **Ohm´s Law** | | **Poiseuille´s Law** | |
| --- | --- | --- | --- |
| ΔVe  Ie =  Re | | ΔPcor ΔPcor  Qcor = =  RCor (8 μ L / π r^4^ ) | |
| Ie | Intensidad de Corriente | Qcor | Flujo Coronario |
| ΔVe | Gradiente de Voltaje Eléctrico | ΔPcor | Gradiente de Presión Transcoronario |
| Re | Resistencia Eléctrica | Rcor | Resistencia Coronaria |

Table 1. Equivalence between Ohm's Law and Poiseuille's Law

Under basal physiological conditions, the relationship between pressure and blood flow in the coronary arteries is not linear, with coronary flow remaining stable over a wide range of pressures. This phenomenon is known as "coronary autoregulation" (8). However, in situations of complete vasodilatation of resistance vessels, either physiologically induced by exercise or pharmacologically induced, for example, with adenosine, the relationship between pressure gradient and coronary flow becomes linear, allowing the application of the consequences of "Poiseuille's law” to obtain coronary physiological indices and thus assess coronary circulatory function (8,10).

**- Coronary Flow Reserve (CFR)**(11)

The increase in flow from the basal situation, where the "coronary autoregulation" mechanism governs, to the situation of maximum hyperemia, in which the coronary flow rate depends on the pressure gradient in the coronary circulation, is an excellent indicator of the functional state of the circulation, both at the macrovascular and microvascular levels. Under normal conditions, coronary circulation can increase more than five times in maximum hyperemia compared to the baseline situation.

Similarly, coronary flow (CF) can be evaluated by thermodilution technique by determining the "Mean Time Transit" (Tmn) by injecting saline at room temperature into the left coronary artery and quantifying the change in temperature of the "blood-saline mixture" between 2 temperature sensors of an intracoronary guidewire positioned distally in the artery to be evaluated. Thus, CF would be the inverse of Tmn, and CFR would correspond to the ratio between CF at maximum hyperemia and CF at rest, which accounts for the capacity of the coronary bed to increase CF in situations of high energy requirements that require an increase in cardiac output and thus the coronary flow necessary for the myocardium to cope with this increase in cardiac output. It is determined as follows (11):

| **Coronary Flow (CF)** | |
| --- | --- |
| 1  CF =  Tmn | |
| **Coronary Flow Reserve (CFR)** | |
| Coronary Flow at Hyperemia 1 / Tmn Hyp Tmn Rest  CFR = = =  Coronary Flow at Rest 1 / Tmn Rest Tmn Hyp | |
| CF | Coronary Flow |
| Tmn | Mean Time Transit |
| CFR | Coronary Flow Reserve |
| Normal value of CFR | ≥ 2.0 |

Table 2. Coronary Flow (CF) and Coronary Flow Reserve (CFR)

**-** **Index of Microvascular Resistance (IMR)**(12)**.**

The IMR is an index developed to evaluate the resistance of the microvascular compartment. Following "Poiseuille's law”, combined with the principle of maximal hyperemia generation, the pressure at the distality of the macrovascular bed and the Tmn at maximal hyperemia are determined.

Based on these assumptions, a coronary index highly related to microvascular dysfunction is obtained as follows.:

| **Coronary Flow at Hyperemia** | | |
| --- | --- | --- |
| 1  Q Hyp =  Tmn Hyp | | |
| **Index of Microvascular Resistance (IMR)** | | |
| ΔPmicrov Hyp P at Distal LAD (Pd) – P Venous Sinus (Pv)  IMR = =  Q Hyp 1 / Tmn Hyp | | Dado que  Pv ≈ 0 |
| IMR = Pd × Tmn Hyp | | |
| Q Hyp | Coronary Flow at Hyperemia | |
| Tmn Hyp | Mean Time Transit at Hyperemia | |
| ΔPmicrov Hyp | Transmicrovascular Gradient at Hyperemia | |
| Normal value of IMR | < 25 | |

Table 3. Index of microvascular resistance.

**- Fractional Flow Reserve (FFR)**(13,14)**.**

The FFR is a coronary hyperemic index that allows a functional evaluation of epicardial lesions, also based on the linear relationship between pressure and flow in the coronary tree during maximum hyperemia.

On this basis, the translesional pressure gradient generated by an epicardial stenosis is proportional to the pressure drop across the lesion. Therefore, an FFR value of 0.60 for an epicardial lesion would correspond to a 40% reduction in coronary flow at maximum hyperemia (or coronary reserve), attributable to that coronary lesion compared to the same artery if it did not present such stenosis. It is an index with a large body of evidence and is the gold standard of coronary indices for determining ischemia related to macrovascular compartment disease. It is determined as follows:

| **Fractional Flow Reserve (FFR)** | | |
| --- | --- | --- |
| At Hyperemia:  P distal to lesion (Pd) - P at Venous Sinus (Pv)  FFR =  P in the aorta (Pa) – P in the venous sinus (Pv) | | Dado que  Pv ≈ 0 |
| At Hyperemia:  Pd  FFR =  Pa | | |
| Normal value of FFR | > 0.80 | |

Table 4. Fractional Flow Reserve (FFR).

**- ‘Resting full cycle ratio’ (RFR) y `Adjusted Resting Full-Cycle Ratio´ (RFRa)**(15)**.**

Given the need to induce maximum hyperemia for the evaluation of FFR using vasodilator drugs that could cause adverse reactions and/or hinder the generalization of physiological assessment techniques for epicardial lesions, resting or nonhyperemic coronary indices were developed for the assessment of the macrovascular compartment. These are indices that do not require drug administration and have a high correlation with FFR values.

Among these indices, the RFR stands out. This is an index that, without the need for vasodilators, allows the hemodynamic significance of epicardial lesions to be assessed by identifying the lowest value of the pressure ratio distal to the stenosis (Pd) / aortic pressure (Pa) during the entire cardiac cycle at rest and, unlike other resting indices, its measurements are less dependent on the morphology of the pressure waves, the electrical signal and the phasic variations in microcirculatory resistance.

However, although the correlation between RFR and FFR is good, a non-negligible percentage of lesions can be misclassified using FFR as a reference. Therefore, in our group, we have developed the RFRa index that allows adjustment for differences based on other clinical and/or angiographic parameters, thus increasing the concordance between techniques.

| **Resting Full-Cycle Ratio (RFR)** | |
| --- | --- |
| Pd  FFR = Lowest value of during whole cardiac cycle  Pa | |
| **Adjusted Resting Full-Cycle Ratio (RFRa)** | |
| RFRa = 0.009 + 0.912*RFR + 0.023*CKD - 0.019*Non-LAD - 0.017*ACS – 0.005*IHD | |
| CKD | Chronic kidney disease |
| Non-LAD | Non-left anterior descending artery lesions |
| ACS | Acute coronary syndrome |
| IHD | Prior ischemic heart disease |
| Normal value of RFR | > 0.89 |
| Normal value of RFRa | > 0.8172 |

Table 5. Resting Full-Cycle Ratio. Adjusted Resting Full-Cycle Ratio.

**- Computational angiographic models for the evaluation of coronary compartments**(16)**.**

# Based on the principles above and computational flow dynamics, computational models have been developed that allow estimation of the involvement of the coronary compartment using angiography-based coronary indices based on angiography. Quantitative Flow Ratio (QFR) is an index that has been shown to correlate well with FFR, being superior to conventional angiography in assessing potential ischemia generated by epicardial coronary lesions. In addition, more recently, coronary indices based on angiography have even been developed that allow the estimation of microvascular resistance without the need to use pressure guides, such as the "Angiography-derived Index of Microcirculatory Resistance" (IMRangio).

**- Acetylcholine coronary vasoreactivity test** (1)**.**

Under normal conditions, an artery with a healthy endothelium responds to acetylcholine with release of nitric oxide, generating vasodilatation. In cases of blocking the action of the enzyme nitric oxide synthase or denuding the arterial wall of the endothelium, vasoconstriction is generated on the contrary, secondary to a stimulation of smooth muscle muscarinic receptors, not counteracted by nitric oxide of endothelial origin. Therefore, acetylcholine infusion can be used to assess endothelial function and adequate coronary vessel tonicity. A relevant percentage of patients with angina without associated obstructive coronary artery disease may present with this type of alteration. Although different methods have been used to reveal this entity, currently the most widely used currently is the intracoronary acetylcholine infusion test. Its objective to determine the presence of endothelial dysfunction or tonic alterations is based on the reproduction of symptoms, the triggering of vasoconstriction and the detection of electrocardiographic alterations. The evaluation of the results of this test is detailed below:

| **Intracoronary Acetylcholine Infusion Test: Diagnostic Criteria** | |
| --- | --- |
| - Negative with vasodilator response (normal):   -Non-clinical  -No ECG changes  -Vasodilation after Ach (with respect to baseline) | - Negative with vasoconstrictor response:   -Non-clinical  -No ECG changes  -No constriction ≥ 90% (with respect to NTG)  -Mild vasoconstriction (with respect to baseline) |
| - Epicardial vasospasm:   -Symptom recurrence  -ST rise or fall  -Constriction ≥ 90% (with respect to NTG) | - Microvascular vasospasm:   -Symptom recurrence  -ST-segment elevation  -No constriction ≥ 90% (with respect to NTG). |

Table 6. Intracoronary acetylcholine infusion test.

**- MicroRNAs and cardiovascular disease**

**- Synthesis and function of microRNAs.**

In the human genome, protein-coding genes represent only 1.1%, whereas more than 70% of the genome is transcribed giving rise to noncoding RNA (nc-RNA) that play important roles in the regulation of pathophysiological processes and cellular homeostasis.

miRNAs are a type of small nc-RNA (17 to 25 nucleotides) that play an important role in the regulation of gene expression. To date, miRNAs are the most studied and described nc-RNAs in the literature (17).

The initial concept that miRNAs regulate gene expression was developed almost 30 years ago, in 1993 in the laboratories of Ambros and Ruvkun when studying the development of the nematode *Caenorhabditis elegans* (18). Several studies have shown that miRNAs are evolutionarily conserved across species and are often ubiquitously expressed (19) (20)(21)

miRNAs can negatively regulate gene expression at the post-transcriptional level by binding to messenger RNA (mRNA) and causing gene silencing by inhibiting translation and/or degradation of mRNA; as well as activating the transcription of certain target genes. MiRNAs have been estimated to control the activity of 30-50% of protein-coding genes, playing an important role in the regulation of several biological processes, including embryogenesis, cell proliferation and differentiation, apoptosis, or tumorogenesis (22).

In the cardiovascular system, miRNAs regulate various processes including cardiomyocyte growth and contractility, heart rhythm development and maintenance, plaque formation, lipid metabolism, and angiogénesis (6). Altered expression of certain miRNAs can be found in blood samples from patients with various cardiovascular diseases, making them attractive candidates as biomarkers.

**- Potential of microRNAs as biomarkers.**

Although miRNAs were initially described as intracellular regulators, they have been stably detected in body fluids. Extracellular microRNAs participate in intercellular communication at the autocrine, paracrine, and endocrine levels and have been implicated in both physiological and adaptive responses, as well as in the emergence and development of disease states. Thus, the concentration and extracellular composition of miRNA reflect pathophysiological states and provide information on the molecular phenotype of the patient under different conditions (23–26).

As reviewed by our group, extracellular miRNAs have the optimal biochemical properties to become excellent biomarkers: (a) they can be obtained by minimally invasive techniques in clinical samples; b) they are highly stable and have a long half-life within the sample; c) they can be quantified by standard techniques already available in clinical laboratories: quantitative reverse transcription PCR (qPCR); d) global profiles could be obtained in a single experiment by RTqPCR, or relatively accessible techniques such as next-generation sequencing or microarrays (27). Based on these characteristics, the circulating miRNA profile constitutes a new generation of biomarkers in various cardiovascular diseases.

**- Relationship between microRNAs and cardiovascular disease: summary of the evidence.**

Currently, many miRNAs are already associated, through different pathophysiological mechanisms, with cardiovascular disease. In the following, we summarize the current evidence on the main miRNAs evaluated in clinical scenarios related to the object of our research, namely atherosclerosis and coronary artery disease.

| **miRNAs and atherosclerosis** | |
| --- | --- |
| miR-126-3p | One of the first miRNAs identified in the development of atherosclerosis.  It is highly expressed in endothelial cells and its expression is essential in vascular development.  Reduced levels favor plaque formation.  Inhibits the expression of VCAM-1, a molecule expressed by endothelial cells that allows the adhesion and migration of leukocytes to the vascular wall(28) |
| miR-21-5p | Related to the early stages of the atherosclerotic process in patients with arterial hypertension (29). |
| miR-155-5p | Expression induced by TNF-α and IFN-β. Increases the population of granulocytes and monocytes during inflammation. Also regulates the expression of the AT1R gene (angiotensin II type 1 receptor) found to be associated with the regulation of blood pressure and atherosclerosis (30). |
| miR-143-5p, miR-145-5p | They are expressed in vascular smooth muscle cells (VSMC) and inhibit migration and proliferation within the developing atherosclerotic plaque (31) |
| miR-133a-5p | Inhibits the growth and proliferation of vascular smooth muscle cells (32) |
| miR-122 -5p | It favors atherosclerosis through the regulation of cholesterol homeostasis. It also promotes inflammation and myocardial fibrosis (33) |
| miR-100-5p | It suppresses the expression of endothelial adhesion molecules attenuating the interaction between the endothelium and leukocytes and reducing inflammation of atheromatous plaques (34) |
| **miRNAs and coronary artery disease** | |
| miR-1-3p, miR-133a-3p, miR-208a-3p, miR-499a-5p | They are overexpressed in patients with acute coronary syndrome and are related to the extent of damage and the prognosis of the patient (35,36). |
| miR-126-3p, miR-17-5p, miR-92a-3p | Infra expression of these miRNAs in patients with stable coronary artery disease compared to healthy subjects (37). |
| miR-126-3p | It has shown a positive association with AMI in the general population in a 10-year follow-up (prospective study) (38) |
| miR-197-3p, miR-223-3p | It has been shown to have a positive association with AMI in the general population (39) |
| miR-126-3p, miR-145-5p | The presence of transcoronary gradients is related to the presence of unstable atheromatous plaques. (40) |
| miR-1-3p | Inhibits Spred1 gene expression and promotes angiogenesis and collateral circulation formation. In relation to cardiogenesis and regulation of stem cell differentiation in cardiomyocytes. (41,42) |
| miR-214-3p | Its levels correlate with the expression of vascular endothelial growth factor. An increase in the concentration of this RNA can be used to predict the presence and severity of coronary lesions in patients with coronary artery disease (43) |
| miR-499a-5p | Highly enriched in the embryonic heart  - Involved in late cardiogenic stages  - Responsible for terminal differentiation of myoblasts to cardiomyocytes and fast/slow muscle fiber specification (44,45) |
| miR-208a-3p | Involved in the late stages of cardiac development.  - Related to the cardiomyocyte involvement of myoblast cardiomyocytes.  - Regulates the expression of cardiac myosin heavy chain (the major contractile protein) (44) |
| miR-142 | Potential marker for predicting MACE in patients with coronary artery disease after percutaneous coronary interventionism.(46) |

Table 7. Main microRNAs associated with cardiovascular disease (CVD) and their mode of action.

**- Variability of microRNAs in published studies.**

As mentioned above, the evidence on miRNAs has increased significantly in recent years. However, it is important to note that the usefulness of miRNAs as biomarkers can vary between studies. Factors such as the clinical setting, the type of sample, as well as the time of collection and its processing, can affect the results reported in different studies (42-48). A recent meta-analysis analyzing and comparing miRNAs isolated in patients with coronary artery disease with respect to healthy subjects showed overexpression of certain miRNAs in patients with coronary artery disease, while other miRNAs showed inconsistent results with overexpression in some studies and underexpression in others (47). This study also shows interesting comparisons between miRNAs isolated in stable coronary artery disease and in acute coronary syndrome compared to healthy controls as shown in the following table.

| Coronary artery disease (any type) versus healthy controls |
| --- |
| Overexpression in coronary artery disease: miR-1-3p, miR-499a-5p, miR-133a-3p, miR-208a-3p/208b-3p, miR-21-5p, miR-142-5p, miR-145-5p |
| Over and under-expressed: miR-126-3p, miR-208b-5p, miR-92a-3p, miR-145-5p y miR-142-5p |
| Stable coronary artery disease versus healthy controls. |
| MiR overexpressed: miR-125a-5p, miR-187-3p, miR 502-5p |
| Underexpressed MiRs: miR-145-5p, miR-29b-3p, let-7 |
| Over- and under-expressed: miR-126-3p, miR-155-5p, miR-17-5p, miR-92a-3p |
| Acute coronary syndrome versus healthy controls. |
| MiR overexpressed: miR-1-3p, miR-499a-5p, miR-208a-3p/208b-3p, miR-133a-3p, miR-133b-3p, miR-27a-3p, miR-30e-5p, miR-93-5p, miR-21-5p |
| Over- and under-expressed: let-7, miR-208b-3p, miR-126-3p, miR-145-5p, miR-134, miR-223-3p, miR-142-5p |
| Acute coronary syndrome versus stable coronary artery disease. |
| Overexpressed in acute coronary syndromes: miR-21, miR-208a-3p/208b-3p, miR-133a-3p, miR-133b-3p, miR-140-5p, miR-146a-5p, miR-499a-5p, miR-27a-3p, miR-27b-3p, miR-451a, miR-29a-3p, miR-29c-3p |
| Over and under-expressed: miR-30a-5p, let-7, miR-142-5p, miR26a-5p, miR26b-5p, miR-92a-3p, miR-150-5p, miR-22-3p, miR-223-3p |

Table 8. MicroRNAs identified in patients with coronary artery disease (stable and/or acute coronary syndrome). In green microRNAs with underexpression. In red microRNAs with overexpression. In yellow microRNAs with over/under expression. (47)

Therefore, despite the large volume of literature on miRNAs in different heart diseases, we must be cautious when interpreting the data offered by the different studies and use a robust methodology that allows a rigorous evaluation of miRNAs in any research project that contemplates the evaluation of these biomarkers.

**HYPOTHESIS**

Different patterns of coronary artery involvement are associated with the overexpression or underexpression of specific miRNAs.

**PRIMARY OBJECTIVE**

Characterization of miRNA expression as a function of the coronary compartment in patients with chest pain.

**SECONDARY OBJECTIVES**

1.- To evaluate the association of miRNA expression with the degree of involvement of the stenosis and the extent of epicardial coronary artery.

2.- To evaluate the association of miRNAs levels with angiography-based indices for determination of coronary compartments.

3.- To evaluate the association of miRNA expression with the presence of cardiovascular risk factors and/or established vascular disease.

4.- To evaluate the association of miRNA expression with other hematological and biochemical markers.

**MATERIAL Y METHODS.**

**- Recruitment.**

Patients with angina referred for coronary angiography at the Universitary Hospital Arnau de Vilanova University Hospital in Lleida will be included. After confirming that the patients are eligible for the study and have signed the informed consent form (**Appendix 1**), the patients will be included in the study.

**- Inclusion criteria:**

1. Age ≥ 18 years.
2. Patients with chest pain suggestive of angina evaluated by a cardiologist referred for diagnostic coronary angiography and eventual coronary angioplasty.
3. Echocardiogram ruling out non-coronary cardiac causes of chest pain.
4. Informed consent.

**- Exclusion criteria:**

1. Contrary to allergy not susceptible to receive pre-medication.
2. Severe bronchial asthma or intolerance to adenosine.
3. Atrioventricular block (≥ 2nd degree) or intolerance to acetylcholine.
4. Acute myocardial infarction with elevation of the ST segment.
5. Acute myocardial infarction without elevation of the ST segment.
6. Cardiogenic shock.
7. Total occlusion of any coronary artery that prevents measurement with pressure-temperature guides.
8. Previous coronary bypass.
9. Women who may be pregnant.
10. Renal dysfunction with estimated glomerular filtration rate < 30 mL/min/1.73m2.
11. Inability to understand the nature of the study and/or sign the informed consent.
12. Any other medical condition that, in the opinion of the investigator, may cause safety problems for the patients or may alter the results of the study.

* NOTE: Given that, in routine clinical practice, a significant proportion of patients are referred for coronary angiography without a previous positive ischemia detection test and even with a negative ischemia detection test in the presence of a high suspicion of anginal pain, in our study the presence of an ischemia detection test is not mandatory for inclusion in the study and it is at the discretion of the referring cardiologist to request coronary angiography.

**- Sample size estimation.**

Based on previous studies of our group and previous studies for the determination of miRNAs in the presence of coronary endothelial dysfunction, we have recruited around 40-50 patients to complete the investigation (7,48–50). In our case, we will study the relationship between miRNAs and the different patterns of involvement according to the affected coronary compartment, that is, Group 1 (Macrovascular and Microvascular Angina); Group 2 (Macrovascular Angina); Group 3 (Microvascular Angina); and Group 4 (Non-coronary chest pain). Given that Group 4 corresponds to patients without coronary circulation alterations, it will be used as a control group with respect to the other groups until recruitment of 25 patients per group is completed, in a similar amount to other control groups in similar studies, totaling 100 patients. Likewise, given the possibility that some patients may present macrovascular and/or microvascular spasms that could overlap with some of the other groups, Group 5 (Macrovascular or Microvascular Spastic Angina) has been defined and will not be included for the purposes of estimating the sample size.

**- Statistical analysis.**

Continuous variables will be expressed as mean ± standard deviation (SD) or median with interquartile range (IQR), according to their distribution. As appropriate, the Mann-Whitney U test will be used to compare continuous variables, while the Chi-square test or Fisher's test will be used to compare categorical variables. If subgroup studies within each group are required (example: patients in group 1 [macrovascular angina and microvascular angina] and diabetes), the Factorial ANOVA test will be performed when equal variances are assumed or the Brown Forsythe test when unequal variances are assumed, followed by post hoc analysis with Bonferroni correction for multiple comparisons. Results will be shown as mean with a 95% confidence interval (CI). The equality of variances between groups will be calculated for each dependent variable using the Leven test, with Bonferroni correction to test for homogeneity of variance between all levels of comparison.

An alpha level of 0.05 will be used to assess statistical significance. Data will be analyzed using SPSS software or STATA.

**- Selection of microRNAs and other samples.**

It is essential to determine those miRNAs that have shown only over- or under-expression in previous studies of coronary atherosclerosis or stable coronary artery disease, i.e:

- Overexpression: miR-1-3p, miR-21-5p, miR-133a-3p, miR-133b-3p, miR-208a-3p,

miR-208b-3p, miR-125a-5p, miR-187-3p, miR-499a-5p, miR-502-5p.

- Under expression: miR-100-5p, miR-143-3p, miR-145-3p, miR-29b-5p.

Furthermore, since miRNA determination kits allow the analysis of multiple miRNAs in each sample, other potential candidates will be determined in agreement with the molecular biology experts. Samples corresponding to routine practice, such as blood counts, biochemistry, lipids, etc., will also be collected.

**- Collection, processing, preservation, and analysis of biological samples.**

In each patient, prior to coronary angiography and the administration of any drug necessary for the coronary angiography, especially heparin required for coronary procedures, as well as iodinated contrast; a 6 mL blood collection (EDTA K2 tube) will be performed. Plasma sample collection and preparation will be performed according to NCI (USA) standard operating procedures. Samples will be stored in the IRBLleida Biobank belonging to the National Biobank Platform. The specific analysis of miRNAs will be performed in the TRRM group laboratory of the Institut de Recerca Biomèdica de Lleida. The analysis of the the rest of biochemical and/or hematological parameters will be performed in the laboratories of the University Hospital Arnau de Vilanova of Lleida. If no differences are observed between miRNAs in the different study groups, the samples will be kept in the IRBLleida Biobank for the assessment of other biomarkers in which the TRRM group has experience, such as lncRNAs, circRNAs, proteins, etc., and for the analysis of the other biomarkers in which the TRRM group has experience, such as lncRNAs, circRNAs, proteins, etc., in the IRBLleida Biobank.

RNA isolation and miRNA quantification will be performed by experienced personnel without access to clinical data. All experiments will be performed using the gold-standard technique; RT-qPCR, under standardized conditions in the same laboratory and according to the previous methodology used by the research group (50–52). Briefly, total RNA will be isolated from 200 μL of frozen plasma samples using the miRNeasy Serum/Plasma Advanced kit (Qiagen) according to the manufacturer's instructions. *Caenorhabditis elegans* miR-39-3p synthetic RNA (cel-miR-39-3p) will be added as external reference RNA. RNA purification will be performed with RNeasy UCP MinElute centrifugation columns according to the manufacturer's recommendations. RNA will be stored in a -80 °C freezer until further analysis. RT-qPCR analysis will be performed according to the recommendations of the MIQE guidelines (53). miRNA quantification will be performed according to the protocol of the miRCURY® LNA® RT Kit (Qiagen) system, which offers optimal accuracy and reproducibility (54). RT reactions will be performed using the miRCURY LNA RT kit (Qiagen). The cDNA will be stored at -20°C. Quantitative PCR (qPCR) will be performed with the miRCURY LNA SYBR® Green PCR kit (Qiagen) using 384-well miRCURY LNA miRNA custom PCR Panels (Qiagen). The qPCR will be performed on a QuantStudio ™ 7 Flex real-time PCR system (Thermo), followed by melting curve analysis. The qPCR amplification curves will be evaluated with QuantStudio Software v1.3 (Thermo). A Cq greater than 35 cycles will be considered undetectable and censored at the minimum level observed for each miRNA. MiRNAs where 80% of the samples meet these criteria will be considered below the detection limit. Relative quantification will be performed using the 2-dCq method, where:

| ΔCq = Cq_miRNA_ − Cq_cel-miR-39-3p_. |
| --- |

**- Bioinformatics analysis.**

It will be performed according to previous publications of the research group (25) . The molecular pathways affected by the identified transcripts will be analyzed using the web-based computational tool DIANA-miRPath v3.0 that combines estimates and experimental data with the KEGG tool to identify molecular pathways.

**- Design**

Illustration 2. Explanatory diagram of the Clinical Phase.

**- Diagnostic coronary angiography and physiological evaluation of the coronary tree.**

After diagnostic coronary angiography, a standardized analysis of coronary lesions in terms of percentage of stenosis and length of the lesion will be performed by quantitative coronary angiography (QCA), in order to avoid variability in visual estimation of coronary lesions. Subsequently, a functional assessment of epicardial lesions with a pressure guidewire will be performed, first with the measurement of nonhyperemic coronary indexes: "Resting full-cycle ratio" (RFR) and RFR adjusted for discordance predictors ("Adjusted RFR), and then by means of the hyperemic coronary indices "Fractional Flow reserve" (FFR), "Coronary Flow Reserve" (CFR) and "Index of Microcirculatory Resistance" (IMR) positioning the pressure guidewire in the distal segment of each artery to be evaluated, being able to interrogate more than one artery in the same patient. Similarly, in patients referred for coronary angiography who have a previous positive ischemia test and whose epicardial arteries are not considered tributary to evaluation, the pressure guide wire will also be placed in the artery corresponding to the territory compatible with the ischemia detected in the test. In the case of coronary lesions not susceptible to physiological evaluation, the pressure guide wire will be positioned in the distal segment of the anterior descending artery, evaluating the "Coronary Flow Reserve" (CFR) and the "Index of Microcirculatory Resistance" (IMR) in the territory of the anterior descending artery. Measurements will be obtained using the ″PressureWire™ X Guidewire 0.014" device (Abbott Vascular Inc., Santa Clara, CA,). For induction of hyperemia, adenosine will be used as a vasodilator drug. In addition, for each lesion the angiography-derived index "Quantitative Flow Ratio" (QFR) and for each artery the "Angiography-derived Index of Microcirculatory Resistance" (IMRangio) will be evaluated.

As for the performance of the coronary vaso-reactivity test, acetylcholine will be used as described above. Given the risk of epicardial spasm in case of macrovascular ischemia derived from epicardial lesions, the acetylcholine test will be performed only in patients who do not present pathological FFR values (> 0.80). More detailed recommendations for the performance of coronary physiology studies are presented in **Annex 2**.

**- Determination of the affected coronary pattern.**

Once the parameters have been obtained, the angina pattern corresponding to each patient will be determined, based on the values of FFR, CFR and IMR, and the treatment corresponding to the findings obtained will be carried out. The decision of medical, percutaneous, or surgical treatment of epicardial coronary lesions leading to ischemia will be made on the basis of FFR.

Macrovascular involvement will be defined when the FFR value is ≤ 0.80 and microvascular involvement when the IMR value is ≥ 25 or the CFR is < 2.0 in the presence of an FFR > 0.80. Angina patterns will depend on the different combinations according to coronary compartment involvement, namely Group 1 (Macrovascular and Microvascular Angina); Group 2 (Macrovascular Angina); Group 3 (Microvascular Angina); and Group 4 (Noncoronary Chest Pain). Since the present protocol also considers an approach to the study of coronary spasm, an additional group will be considered, through the evaluation of the acetylcholine test in patients presenting microvascular angina or noncoronary chest pain (Groups 3 and 4). Patients presenting macro or microvascular spasm in this test will be included in Group 5 (Macro or Microvascular Spastic Angina).

**SCIENTIFIC INTEREST OF THE RESEARCH.**

The present research proposal could provide relevant results on the epigenomic characterization of angina pectoris by determining miRNAs.

Coronary artery disease is traditionally understood as a phenomenon that begins with endothelial dysfunction and, as it progresses, progressively affects the entire coronary tree (55). Likewise, this process is considered to be delayed for a long period of time, which means that patients with coronary artery disease can present a very prolonged preclinical phase until the development of myocardial ischemia. However, once this phase of the disease has been reached, one of the main prognostic factors is the presence of ischemia. It is also worth highlighting the heterogeneity of the presentation of ischemic heart disease, with data showing that patients can have isolated microvascular or macrovascular involvement, as well as a combination of these (56).

To date, only a recent Spanish article (57) showing the usefulness of miRNAs to distinguish acute myocarditis from acute coronary syndrome has truly awakened the interest of clinical cardiologists in these promising disease markers in other types of pathologies. In relation to coronary artery disease, with the exception of the aforementioned work, the clinical relevance of miRNAs continues to be scarce. This may be due to the fact that the discovery of new markers that provide additional clinical information to that provided by clinical, biochemical or diagnostic imaging factors is difficult given the great development of research in these fields over the last several decades, especially in patients who survive acute myocardial infarction.

In our opinion, the great a priori strength of this investigation would lie in the robust determination of four comparison groups, with the most specific techniques for their characterization (coronary physiology studies), which would consider the three possible combinations of involvement of the compartments of the coronary tree (Groups 1 to 3) and a group of patients without ischemic heart disease (Group 4). Likewise, a final group (Group 5) would provide additional information on the presence of coronary spasm. The precise definition of these groups would allow a robust assessment of over or under-expression, and even the existence of gradients in miRNAs depending on the extent of myocardial ischemia and the number of coronary vascular compartments affected.

So far, few papers have evaluated miRNAs in a manner similar to our research proposal, such as in coronary endothelial dysfunction (7). However, to our knowledge, the role of miRNAs in relation to microvascular dysfunction such as IMR has not been specifically evaluated, nor has a study been developed that attempts to globally understand the role of miRNAs in relation to the coronary vascular compartments involved in the presence of myocardial ischemia.

It is also noteworthy that research on coronary physiology is experiencing a new boom, and in our opinion, contributions in this field would be very valuable. In addition, the determination of miRNA patterns could provide an opportunity to improve patient treatment and even reduce the need for invasive physiological tests. For all these reasons, we consider that the present study could be of great scientific interest.

In addition, a contingency plan will be established in the event that differences between miRNAs in the different study groups, since we will have a sample bank that will allow us to analyze other biomarkers where the research group has experience. Likewise, a dissemination plan aimed at the general public will be established, and actions will be carried out to inform segments of the population of special interest/vulnerability within the scope of the study. The information obtained will be made available to the scientific world and the general public in collaboration with the IRBLleida Communication Department. IRBLleida has taken several steps to disseminate scientific messages in a clear and understandable way. We will use the IRBLleida website (https://www.irblleida.org/es/) that contains information on projects, publications and research groups. To make research more accessible to society, we will use digital communication platforms. Additionally, some of the researchers are members of the European COST CardioRNA program (https://cardiorna.eu/). One of the working groups of this program is focused purely on the communication of the results obtained.

**ANALYSIS OF PROJECT FEASIBILITY.**

We consider the project to be viable for the following reasons. The Hemodynamics Unit of the University Hospital Arnau de Vilanova of Lleida performs more than 1,110 diagnostic coronary angiograms and more than 500 coronary angioplasties per year and, therefore, invasive coronary physiology studies could be carried out within a reasonable time period.

Similarly, the Institut de Recerca Biomèdica de Lleida has the necessary technology to carry out the processing, preservation of samples, and analysis of miRNAs, with extensive experience and publications in this field. In addition, at the Cardiology Service of the University Hospital Arnau de Vilanova of Lleida we have a Research Unit with a nurse specifically dedicated to this task, which ensures the protocolized extraction of sample.

It should also be noted that the Cardiology Department of the Arnau de Vilanova has experience in this field, as shown by the fact that it has conducted research on the validation and development of nonhyperemic coronary indexes (58), as well as pathologies presenting microvascular dysfunction, such as TakoTsubo syndrome (59).

**REFERENCES.**

1. Knuuti J, Winjs W, Saraste A, Capodanno D, Barbato E, Funck-Brentano C, et al. 2019 ESC Guidelines for the diagnosis and management of chronic coronary syndromes. Eur Heart J. 2020 Jan 14;41(3):407–77.

2. Kunadian V, Chieffo A, Camici PG, Berry C, Escaned J, Maas AHEM, et al. An EAPCI Expert Consensus Document on Ischaemia with Non-Obstructive Coronary Arteries in Collaboration with European Society of Cardiology Working Group on Coronary Pathophysiology & Microcirculation Endorsed by Coronary Vasomotor Disorders International. European Heart Journal. 2020;41(37):3504–20.

3. Candell-Riera J, Martin-Comín J, Escaned J, Peteiro J. Physiologic evaluation of coronary circulation. Role of invasive and non invasive techniques. Revista Espanola de Cardiologia. 2002;55(3):271–91.

4. Rahman H, Corcoran D, Aetesam-Ur-Rahman M, Hoole SP, Berry C, Perera D. Diagnosis of patients with angina and non-obstructive coronary disease in the catheter laboratory. Heart. 2019 Oct 1;105(20):1536–42.

5. Ford TJ, Stanley B, Good R, Rocchiccioli P, McEntegart M, Watkins S, et al. Stratified Medical Therapy Using Invasive Coronary Function Testing in Angina: The CorMicA Trial. J Am Coll Cardiol. 2018 Dec 11;72(23 Pt A):2841–55.

6. A H. Functions of microRNAs in cardiovascular biology and disease. Annu Rev Physiol. 2013 Feb 10;75:69–93.

7. Widmer RJ, Chung WY, Herrmann J, Jordan KL, Lerman LO, Lerman A. The association between circulating microRNA levels and coronary endothelial function. PLoS One [Internet]. 2014 Oct 13 [cited 2022 Jan 28];9(10). Available from: https://pubmed.ncbi.nlm.nih.gov/25310838/

8. Rubio R, Berne RM. Regulation of coronary blood flow. Progress in Cardiovascular Diseases. 1975 Sep 1;18(2):105–22.

9. Hirshfeld JW, Nathan AS. Deriving Function From Structure: Applying Hagen-Poiseuille to Coronary Arteries. JACC: Cardiovascular Interventions. 2020 Feb 24;13(4):498–501.

10. De Bruyne B, Bartunek J, Sys SU, Heyndrickx GR. Relation between myocardial fractional flow reserve calculated from coronary pressure measurements and exercise-induced myocardial ischemia. Circulation. 1995 Jul 1;92(1):39–46.

11. Pijls NHJ, De Bruyne B, Simith L, Aarnoudse W, Barbato E, Bartunek J, et al. Coronary thermodilution to assess flow reserve: validation in humans. Circulation. 2002 May 28;105(21):2482–6.

12. Fearon WF, Balsam LB, Farouque HMO, Robbins RC, Fitzgerald PJ, Yock PG, et al. Novel index for invasively assessing the coronary microcirculation. Circulation. 2003 Jul 1;107(25):3129–32.

13. Pijls NHJ, Van Son JAM, Kirkeeide RL, De Bruyne B, Gould KL. Experimental basis of determining maximum coronary, myocardial, and collateral blood flow by pressure measurements for assessing functional stenosis severity before and after percutaneous transluminal coronary angioplasty. Circulation. 1993;87(4):1354–67.

14. Pijls NHJ, De Bruyne B, Peels K, Van Der Voort PH, Bonnier HJRM, Bartunek J, et al. Measurement of fractional flow reserve to assess the functional severity of coronary-artery stenoses. N Engl J Med. 1996 Jun 27;334(26):1703–8.

15. Svanerud J, Ahn JM, Jeremias A, Van ’T Veer M, Gore A, Maehara A, et al. Validation of a novel non-hyperaemic index of coronary artery stenosis severity: The Resting Full-cycle Ratio (VALIDATE RFR) study. EuroIntervention. 2018 Sep 1;14(7):806–14.

16. Scarsini R, Shanmuganathan M, de Maria GL, Borlotti A, Kotronias RA, Burrage MK, et al. Coronary Microvascular Dysfunction Assessed by Pressure Wire and CMR After STEMI Predicts Long-Term Outcomes. JACC Cardiovascular Imaging [Internet]. 2021 Apr 14 [cited 2022 Mar 3];14(10):1948–59. Available from: https://europepmc.org/article/MED/33865789

17. Çakmak HA, Demir M. Microrna and cardiovascular diseases. Balkan Medical Journal. 2020;37(2):60–71.

18. Lee RC, Feinbaum RL, Ambros V. The C. elegans heterochronic gene lin-4 encodes small RNAs with antisense complementarity to lin-14. Cell [Internet]. 1993 Dec 3 [cited 2022 Jan 24];75(5):843–54. Available from: https://pubmed.ncbi.nlm.nih.gov/8252621/

19. Lagos-Quintana M, Rauhut R, Lendeckel W, Tuschl T. Identification of novel genes coding for small expressed RNAs. Science [Internet]. 2001 Oct 26 [cited 2022 Jan 24];294(5543):853–8. Available from: https://pubmed.ncbi.nlm.nih.gov/11679670/

20. Lau NC, Lim LP, Weinstein EG, Bartel DP. An abundant class of tiny RNAs with probable regulatory roles in Caenorhabditis elegans. Science [Internet]. 2001 Oct 26 [cited 2022 Jan 24];294(5543):858–62. Available from: https://pubmed.ncbi.nlm.nih.gov/11679671/

21. Lee RC, Ambros V. An extensive class of small RNAs in Caenorhabditis elegans. Science [Internet]. 2001 Oct 26 [cited 2022 Jan 24];294(5543):862–4. Available from: https://pubmed.ncbi.nlm.nih.gov/11679672/

22. Wojciechowska A, Braniewska A, Kozar-Kamińska K. MicroRNA in cardiovascular biology and disease. Advances in Clinical and Experimental Medicine. 2017;26(5):865–74.

23. Hergenreider E, Heydt S, Tréguer K, Boettger T, Horrevoets AJG, Zeiher AM, et al. Atheroprotective communication between endothelial cells and smooth muscle cells through miRNAs. Nature Cell Biology 2012 14:3 [Internet]. 2012 Feb 12 [cited 2022 Apr 8];14(3):249–56. Available from: https://www.nature.com/articles/ncb2441

24. Oerlemans MIFJ, Mosterd A, Dekker MS, de Vrey EA, van Mil A, Pasterkamp G, et al. Early assessment of acute coronary syndromes in the emergency department: the potential diagnostic value of circulating microRNAs. EMBO Mol Med [Internet]. 2012 Nov [cited 2022 Apr 8];4(11):1176–85. Available from: https://pubmed.ncbi.nlm.nih.gov/23023917/

25. de Gonzalo-Calvo D, Dávalos A, Montero A, García-González Á, Tyshkovska I, González-Medina A, et al. Circulating inflammatory miRNA signature in response to different doses of aerobic exercise. J Appl Physiol (1985) [Internet]. 2015 Jul 15 [cited 2022 Mar 5];119(2):124–34. Available from: https://pubmed.ncbi.nlm.nih.gov/25997943/

26. Pijls NHJ, de Bruyne B, Smith L, Aarnoudse W, Barbato E, Bartunek J, et al. Coronary Thermodilution to Assess Flow Reserve. Circulation. 2002 May 28;105(21).

27. Calderon-Dominguez M, Belmonte T, Quezada-Feijoo M, Ramos-Sánchez M, Fernández-Armenta J, Pérez-Navarro A, et al. Emerging role of microRNAs in dilated cardiomyopathy: evidence regarding etiology. Translational Research [Internet]. 2020 Jan 1 [cited 2022 Apr 8];215:86–101. Available from: http://www.translationalres.com/article/S1931524419301707/fulltext

28. Harris TA, Yamakuchi M, Ferlito M, Mendell JT, Lowenstein CJ. MicroRNA-126 regulates endothelial expression of vascular cell adhesion molecule 1. Proc Natl Acad Sci U S A. 2008 Feb 5;105(5):1516.

29. Cengiz M, Yavuzer S, Avcı BK, Yürüyen M, Yavuzer H, Dikici SA, et al. Circulating miR-21 and eNOS in subclinical atherosclerosis in patients with hypertension. https://doi.org/103109/1064196320151036064. 2015 Nov 17;37(8):643–9.

30. Faraoni I, Antonetti FR, Cardone J, Bonmassar E. miR-155 gene: A typical multifunctional microRNA. Biochimica et Biophysica Acta (BBA) - Molecular Basis of Disease. 2009 Jun 1;1792(6):497–505.

31. Cordes KR, Sheehy NT, White M, Berry E, Morton SU, Muth AN, et al. miR-145 and miR-143 Regulate Smooth Muscle Cell Fate Decisions. Nature. 2009 Aug 6;460(7256):705.

32. Torella D, Laconetti C, Catalucci D, Ellison G, Leone A, Waring C, et al. MicroRNA-133 controls vascular smooth muscle cell phenotypic switch in vitro and vascular remodeling in vivo. Circ Res. 2011 Sep 30;109(8):880–93.

33. Liu Y, Song J-W, Lin J-Y, Miao R, Zhong J-C. Roles of MicroRNA-122 in Cardiovascular Fibrosis and Related Diseases. Cardiovascular Toxicology. 2020 Oct 1;20(5):1.

34. Pankratz F, Hohnloser C, Bemtgen X, Jaenich C, Kreuzaler S, Hoefer I, et al. MicroRNA-100 Suppresses Chronic Vascular Inflammation by Stimulation of Endothelial Autophagy. Circulation Research. 2018 Feb 2;122(3):417–32.

35. S DR, F E, C C, A S, G A, J S, et al. Transcoronary concentration gradients of circulating microRNAs in heart failure. Eur J Heart Fail. 2018 Jun 1;20(6):1000–10.

36. Eitel I, Adams V, Dieterich P, Fuernau G, De Waha S, Desch S, et al. Relation of circulating MicroRNA-133a concentrations with myocardial damage and clinical prognosis in ST-elevation myocardial infarction. American Heart Journal. 2012 Nov 1;164(5):706–14.

37. Fichtlscherer S, De Rosa S, Fox H, Schwietz T, Fischer A, Liebetrau C, et al. Circulating microRNAs in patients with coronary artery disease. Circ Res. 2010 Sep 3;107(5):677–84.

38. Zampetaki A, Willeit P, Tilling L, Drozdov I, Prokopi M, Renard JM, et al. Prospective Study on Circulating MicroRNAs and Risk of Myocardial Infarction. J Am Coll Cardiol. 2012 Jul 24;60(4):290–9.

39. Wang G-K, Zhu J-Q, Zhang J-T, Li Q, Li Y, He J, et al. Circulating microRNA: a novel potential biomarker for early diagnosis of acute myocardial infarction in humans. European Heart Journal. 2010 Mar 1;31(6):659–66.

40. Leistner DM, Boeckel J-N, Reis SM, Thome CE, De Rosa R, Keller T, et al. Transcoronary gradients of vascular miRNAs and coronary atherosclerotic plaque characteristics. European Heart Journal. 2016 Jun 7;37(22):1738–49.

41. J L, X D, Z W, J W. MicroRNA-1 in Cardiac Diseases and Cancers. The Korean Journal of Physiology & Pharmacology : Official Journal of the Korean Physiological Society and the Korean Society of Pharmacology. 2014 Oct 17;18(5):359–63.

42. Deng F, Xu X, Chen Y-H. The Role of miR-1 in the Heart: From Cardiac Morphogenesis to Physiological Function. Human Genetics & Embryology. 2014;4(1):1–4.

43. Jin Y, Yang C-J, Xu X, Cao J-N, Feng Q-T, Yang J. MiR-214 regulates the pathogenesis of patients with coronary artery disease by targeting VEGF. Molecular and Cellular Biochemistry 2015 402:1. 2015 Jan 10;402(1):111–22.

44. Chistiakov DA, Orekhov AN, Bobryshev Y V. Cardiac-specific miRNA in cardiogenesis, heart function, and cardiac pathology (with focus on myocardial infarction). Journal of Molecular and Cellular Cardiology. 2016 May 1;94:107–21.

45. Espinoza-Lewis RA, Wang DZ. MicroRNAs in Heart Development. Current Topics in Developmental Biology. 2012 Jan 1;100:279–317.

46. Tang Q, Lei H, Wu H, Chen J, Deng C, Sheng W, et al. Plasma miR-142 predicts major adverse cardiovascular events as an intermediate biomarker of dual antiplatelet therapy. Acta Pharmacologica Sinica. 2019 Feb 1;40(2):208.

47. Kaur A, Mackin ST, Schlosser K, Wong FL, Elharram M, Delles C, et al. Systematic review of microRNA biomarkers in acute coronary syndrome and stable coronary artery disease. Cardiovascular Research. 2021;116(6):1113–24.

48. de Gonzalo-Calvo D, Vilades D, Martínez-Camblor P, Vea À, Nasarre L, Sanchez Vega J, et al. Circulating microRNAs in suspected stable coronary artery disease: A coronary computed tomography angiography study. J Intern Med [Internet]. 2019 [cited 2022 Apr 8];286(3):341–55. Available from: https://pubmed.ncbi.nlm.nih.gov/31141242/

49. de Gonzalo-Calvo D, Martínez-Camblor P, Bär C, Duarte K, Girerd N, Fellström B, et al. Improved cardiovascular risk prediction in patients with end-stage renal disease on hemodialysis using machine learning modeling and circulating microribonucleic acids. Theranostics [Internet]. 2020 [cited 2022 Apr 8];10(19):8665–76. Available from: https://pubmed.ncbi.nlm.nih.gov/32754270/

50. Vilades D, Martínez-Camblor P, Ferrero-Gregori A, Bär C, Lu D, Xiao K, et al. Plasma circular RNA hsa_circ_0001445 and coronary artery disease: Performance as a biomarker. FASEB J [Internet]. 2020 Mar 1 [cited 2022 Mar 5];34(3):4403–14. Available from: https://pubmed.ncbi.nlm.nih.gov/31999007/

51. de Gonzalo-Calvo D, Vilades D, Martínez-Camblor P, Vea À, Nasarre L, Sanchez Vega J, et al. Circulating microRNAs in suspected stable coronary artery disease: A coronary computed tomography angiography study. J Intern Med [Internet]. 2019 [cited 2022 Mar 5];286(3):341–55. Available from: https://pubmed.ncbi.nlm.nih.gov/31141242/

52. de Gonzalo-Calvo D, Vilades D, Martínez-Camblor P, Vea À, Ferrero-Gregori A, Nasarre L, et al. Plasma microRNA Profiling Reveals Novel Biomarkers of Epicardial Adipose Tissue: A Multidetector Computed Tomography Study. J Clin Med [Internet]. 2019 Jun 1 [cited 2022 Mar 5];8(6). Available from: https://pubmed.ncbi.nlm.nih.gov/31159404/

53. Bustin SA, Benes V, Garson JA, Hellemans J, Huggett J, Kubista M, et al. The MIQE guidelines: minimum information for publication of quantitative real-time PCR experiments. Clin Chem [Internet]. 2009 Apr 1 [cited 2022 Mar 5];55(4):611–22. Available from: https://pubmed.ncbi.nlm.nih.gov/19246619/

54. Mestdagh P, Hartmann N, Baeriswyl L, Andreasen D, Bernard N, Chen C, et al. Evaluation of quantitative miRNA expression platforms in the microRNA quality control (miRQC) study. Nat Methods [Internet]. 2014 [cited 2022 Mar 5];11(8):809–15. Available from: https://pubmed.ncbi.nlm.nih.gov/24973947/

55. Sitia S, Tomasoni L, Atzeni F, Ambrosio G, Cordiano C, Catapano A, et al. From endothelial dysfunction to atherosclerosis. Autoimmunity Reviews. 2010 Oct 1;9(12):830–4.

56. Severino P, D’Amato A, Pucci M, Infusino F, Adamo F, Birtolo LI, et al. Ischemic Heart Disease Pathophysiology Paradigms Overview: From Plaque Activation to Microvascular Dysfunction. International Journal of Molecular Sciences. 2020 Nov 1;21(21):1–30.

57. Blanco-Domínguez R, Sánchez-Díaz R, Fuente H de la, Jiménez-Borreguero LJ, Matesanz-Marín A, Relaño M, et al. A Novel Circulating MicroRNA for the Detection of Acute Myocarditis. https://doi.org/101056/NEJMoa2003608. 2021 May 26;384(21):2014–27.

58. Casanova-Sandoval J, Fernández-Rodríguez D, Otaegu I, Jiménez TG, Rodríguez-Esteban M, Rivera K, et al. Usefulness of the Hybrid RFR-FFR Approach: Results of a Prospective and Multicenter Analysis of Diagnostic Agreement between RFR and FFR-The RECOPA (REsting Full-Cycle Ratio Comparation versus Fractional Flow Reserve (A Prospective Validation)) Study. J Interv Cardiol [Internet]. 2021 [cited 2022 Mar 3];2021. Available from: https://pubmed.ncbi.nlm.nih.gov/34007248/

59. Uribarri A, Nunez-Gil IJ, Conty DA, Vedia O, Almendro-Delia M, Cambra AD, et al. Short- and Long-Term Prognosis of Patients With Takotsubo Syndrome Based on Different Triggers: Importance of the Physical Nature. J Am Heart Assoc [Internet]. 2019 Dec 17 [cited 2022 Mar 3];8(24). Available from: https://pubmed.ncbi.nlm.nih.gov/31830875/

60. Gutiérrez E, Gómez-Lara J, Escaned J, Cruz I, Ojeda S, Romaguera y R, et al. <i class="fa fa-video-camera" aria-hidden="true"></i> Valoraci�n de la funci�n endotelial y provocaci�n de vasoespasmo coronario mediante infusi�n intracoronaria de acetilcolina. Documento t�cnico de la ACI-SEC. REC: interventional cardiology. 2021 Nov 16;

**Annex 1: Informed consent.**

**PATIENT INFORMATION SHEET**

**Study "Epigenetic characterization of angina pectoris according to the coronary compartment affected: relationship between invasive physiological coronary assessment and microRNAs"**

Please carefully read the information provided in this document, ask your doctor any doubts, and ask for any clarification you consider necessary so that you can decide freely and with the necessary information whether or not you want to participate in this research.

**Introduction and objectives:**

Your participation is requested in this research project whose objective is to determine the association of miRNA expression as a function of the affected coronary compartment; in patients suffering from angina pectoris and undergoing cardiac catheterization.

Lesions in the heart arteries (coronary lesions) can limit the amount of blood reaching the heart muscle and cause symptoms such as chest pain (angina pectoris), shortness of breath, and eventually myocardial infarction. Heart artery disease is known to be present in the main arteries (epicardial) or in the microscopic arteries arising from the main arteries (microcirculation). The complete assessment of this disease is performed by cardiac catheterization, which is an invasive test that requires arterial puncture and can present, although not frequently, potential complications.

MicroRNAs are molecules that regulate certain physiological processes, are released into the blood by cells and whose concentration in the blood varies for various reasons, heart artery disease being one of them. Currently, the precise study of heart artery disease requires cardiac catheterization, which, as mentioned above, is an invasive procedure.

MicroRNAs modify their concentration in blood due to lesions in the heart arteries; and are a potential tool for diagnosing this disease in a much simpler, faster, safer and more comfortable way for the patient, since only a blood extraction is required to perform the study. However, this is a novel technique and it is not yet known exactly which microRNAs modify their concentration in blood in patients with coronary lesions.

This study aims to determine which microRNAs modify their concentration in patients with coronary lesions, as well as to determine whether there are specific patterns that allow us to distinguish whether there is disease in the epicardial arteries or in the microcirculation, so that this technique can be standardized and used in the future on a daily basis in routine clinical practice.

This study is being evaluated by the Medical Research Ethics Committee (CEIM) of the Arnau de Vilanova University Hospital of Lleida and complies with the Law 14/2007 on requirements of the Biomedical Research.

**Risks:**

Participation in the study will not imply any inconvenience at a personal level, since the studies to be performed will be exactly the same, whether you accept to participate in the study or not. The only difference that would exist in the event that we accept participation in the study would be that a blood extraction would be performed (without requiring an additional puncture), which would be analyzed and whose results together with anonymized data would be included in a database, in order to be able to perform the study.

**Benefits:**

You will not derive any benefit from your participation in this study. However, investigation of this entity may have diagnostic implications for patients who are at risk or develop it in the future.

**Protection of personal data:**

The confidentiality and protection of the data obtained is guaranteed in accordance with the legislation 2016/679 of the European Parliament and of the Data Protection Council (RGPD) of April 27, 2016 Data Protection (RGPD); and in accordance with the Organic Law 3/2018, of December 5, on the Protection of Personal Data and Guarantee of Digital Rights (LOPD). In accordance with the current law, you have the right to access your personal data; likewise, and if justified, you have the right to rectify and cancel it. If you so wish, you must request it to the Data Protection Delegate by email dpd@ticsalutsocial.cat.

By signing this document, you expressly consent that both the center and the researcher are, respectively, responsible for the processing of your data and undertake to comply with the data protection regulations in force.

Access to the information will be restricted to the personnel carrying out the research, being obliged to maintain the confidentiality of the data. The results of the research may be communicated to the health authorities and the scientific community through congresses and publications.

**Study activities:**

- Your physician will collect your medical history at the time of cardiac catheterization.
- Blood draw during cardiac catheterization without requiring an additional puncture.
- You will not have to come to any additional visits.
- No action different from the usual clinical practice will be performed.

**Biological samples and associated information: in no case will any experimental test be performed.**

During one of the blood extractions that will be performed during the assistance process, 10 additional ml of blood will be collected for the biomedical research studies framed within the present research project. This will not cause you any additional inconvenience. The samples and the information associated with them will be kept in the hospital where you are treated, under the conditions and guarantees of quality and safety required by current legislation. The analysis of the samples will be carried out in the specialized departments of the hospitals involved in this study.

Dr. Lucía Matute Blanco, as coordinator of the program, and the physician responsible for your treatment will make available to you all the information related to the research projects in which the blood samples collected during your participation in the study will be used. The process is always carried out under the supervision of the hospital. The use of the biological sample for a purpose other than that agreed upon in this document will have to be expressly authorized by you in a new consent document.

**Altruistic nature of the donation. The transfer of biological samples that you make is free of charge.**

You will not receive financial benefit for your participation in the research studies or any benefit derived from the discoveries that may be made in this biomedical research.

**Voluntary participation**

Your participation in the study is completely voluntary and you may refuse to participate without explanation. We also inform you that you will be treated the same way and with the same professional rigour regardless of your participation in this study.

**Revocation of consent: If you choose to sign this consent, you may also freely cancel it. This will result in the destruction of your samples.**

If in the future you would like to withdraw your consent, your biological samples would be destroyed and the data associated with them would be removed. However, the effects of this cancellation could not be retrospectively extended to research that has already been carried out. The rights of access, rectification, cancellation and opposition can be exercised before:

Project Coordinator: Lucía Matute Blanco

Arnau de Vilanova University Hospital.

Postal address: Avenida Rovira Roure 80, CP 25198, Lleida, Spain.

Contact telephone number: 973-468024

**Fate of samples after their use in this research project.**

At the end of the research project, leftover samples will be disposed of.

**Contact person:**

You may ask any questions you have during the course of this investigation. If you have any problems or further questions about it or about your rights as a patient, please contact the person listed below

Responsible Physician:

Dr ……………………..…………………………..…………………………..……

Address: …………………..……………………..…………………………..……

Phone: …………………………………………..…………………………..……

**INFORMED CONSENT FORM**

This sheet is specific to participate in the project entitled: **"Epigenetic characterization of angina pectoris according to the affected coronary compartment: relationship between invasive physiological coronary assessment and microRNAs”**

Patient's first and last name: …………………………………………………

Date of Birth: …………………………………………………………….

ID card: ……………………………………………………………………………..

Center: …………………………………………………………………………….

If you have understood the information provided to you, have resolved any doubts you may have and decide to collaborate with the present research project under the terms explained above, please read and sign this sheet below:

The undersigned authorizes the responsible investigator to store and scientifically use both the clinical-assistance information of his/her medical history and the biological material such as the imaging tests that have been or will be performed on him/her, in order to carry out the present research project.

I confirm that:

1. I authorize that surplus biological material used for diagnostic testing and associated clinical information be used for research associated with the above research project: YES NO

2. I wish to receive information derived from research that is truly relevant and applicable to my health: YES NO

3. I authorize to be contacted in case I need further information or additional biological samples: YES NO

Also, I agree with the following points:

1. I have read the study information sheet

2. I have received the necessary information.

3. I have spoken to Dr…………………………………………………………….

and I have had the chance to ask about the project.

4. My participation is voluntary.

5. Participation will not have an impact on the health care you need.

6. All clinical data, test results and my identity cannot be disclosed without my permission.

Signed by (patient): Signed by (Investigator)

_____________________ _______________________

Date: Date:

**Anex 2: Recommendations to perform invasive physiological evaluation**(16,58,60)

| **First, RFR, FFR, CFR and IMR measurements will be performed as detailed below.** |
| --- |
| **Catheter Selection and Positioning**  • Use guide catheters (at least 5F) without lateral holes.  • Ensure coaxial cannulation of the guiding catheter in the coronary ostium.  • Decannulate the guiding catheter from the ostium to calibrate pressure, equalize, and record Pd / Pa if there is any doubt that the catheter may partially obstruct the ostium. |
| **Calibration**  • Before starting measurements, ensure that the aortic pressure is set to zero correctly (1/3 versus 2/3 chest diameter).  • Purge the pressure guide, place it in a horizontal position at the time of connection/calibration and do not move the pressure guide while performing the process.  • Before adjusting pressures, advance the guidewire until the pressure sensor is positioned exactly at the end of the guide catheter.  • Before equalizing the pressures, flush the guiding catheter with saline to remove the viscous contrast agent.  • Before equalizing pressures, remove the introducer and close the hemostatic valve.  • Pressure curves are usually averaged over three to five heartbeats. Therefore, the pressure equalization requires some time and artifacts should not occur during this time. |
| **Positioning of the Pressure-Temperature Guide**  • For the evaluation of epicardial lesions (RFR and FFR), the pressure sensor should be placed distal to the main vessel to be analyzed.  • RFR is measured first, followed by FFR, CFR, and IMR.  • For the determination of CFR and IMR, the pressure sensor should be placed in the distal segment of the vessel corresponding to the lesion to be evaluated.  • In the case of patients referred for coronary angiography who present a positive ischemia test, FFR, CFR and IMR will also be measured, in addition to the arteries of the lesions to be evaluated, in the arteries compatible with the ischemia detected in the ischemia test. Likewise, in the absence of lesions to be evaluated and a previous positive ischemia test to guide the exploration, the FFR, CFR and IMR will be evaluated in the anterior descending artery.  • A second guidewire, in addition to the pressure guidewire, could cause artifacts and should therefore be avoided.  • Detect artifacts: the sensor could interact with the wall of the vessel, especially in cases of narrow vessel caliber or severe tortuosity.  • Viscous contrast agent in the coronary artery may affect the pd/pa gradient. |
| **Hyperemia**  • Before advancing the pressure wire, administer nitroglycerin ic (usually 200 mcg) to prevent coronary spasm.  • Medications for hyperemia.  - Intravenous adenosine 140 μg / kg / min.  • In case of measurement results in the borderline area, an increase in the dose of ev adenosine is possible. However, intravenous doses> 180 μg / kg / min may reduce coronary perfusion and are therefore not recommended. |
| **Recording and Evaluation**  • Artifacts should be carefully observed and excluded.  RFR and FFR  • For the determination of RFR, a minimum of 5 consecutive cardiac cycles are required.  • After administration of intravenous adenosine, pressure values may decrease to a minimum before reaching steady state. Wait until stable FFR values are obtained.  • Values measured during atrioventricular block/adenosine-induced bradycardia should be classified as "not evaluable".  • Ectopic beats may falsify the values obtained, so be sure to obtain values during periods of electrically stable time.  CFR and IMR  • For the determination of CFR and IMR, 3 rapid injections of 3 cc of physiological saline will be made at baseline. Subsequently, during the administration of ev adenosine and once the state of maximum hyperemia is reached, 3 rapid injections of 3cc of physiological saline will be performed again.  • Alterations in the saline temperature or the saline injection rate may cause abnormal Tmn values that are detected by the software. Carefully evaluate the concordance of these values to assess whether any injection should be repeated.  • To determine the CFR, the Tmn information obtained at baseline and at maximum hyperemia is used. To determine the IMR, only the Tmn information obtained at maximum hyperemia is used. |
| **Then, QFR and IMRangio measurements will be performed according to the specifications detailed in the specific software.** |
| **Acetylcholine test will only be performed for patients in Groups 3 and 4 as detailed below.** |
| • Electrocardiographic monitoring of the patient by 12-lead electrocardiogram.  • Cinefluoroscopy images will be obtained in a cranial projection that adequately displays the anterior descending artery.  • Given the expected diffuse nature of coronary spasm, it will be performed by increasing injections of acetylcholine up to 3 doses of 2, 20 and 100 mcg in the left coronary artery.  • Acetylcholine injections will not be performed in the right coronary heart due to the very high risk of severe bradycardia.  • The intracoronary bolus administration time will be performed slowly for 20 seconds.  • The guiding catheter should then be slowly flushed with saline to prevent abrupt injections of the drug remaining in the catheter at the time of acquisition of the cinefluoroscopy image.  • After each injection, the presence of symptoms suggestive of angina and similar to those that motivated the study will be evaluated, a cinefluoroscopy image will be acquired and a 12-lead electrocardiogram will be performed.  • If significant angiographic spasm or ST elevation/decrease suggestive of microvascular or macrovascular spasm is documented, progression to the next dose will not continue.  • In case of significant spasm, as well as at the end of the test, 200 mcg of NTG ic will be administered to abolish the effect of acetylcholine. |
