# Supplementary material for: Study protocol for the epigenetic characterization of angor pectoris according to the affected coronary compartment: Global and comprehensive assessment of the relationship between invasive coronary physiology and microRNAs
Source: PLoS One. 2023 May 11;18(5):e0283097. doi: 10.1371/journal.pone.0283097 (PMC10174526; doi:10.1371/journal.pone.0283097)
Supplement: S5 File — (DOCX) [file pone.0283097.s006.docx]

**Caracterización epigenética de la angina pectoris según el compartimiento coronario afectado: Relación entre la evaluación coronaria fisiológica invasiva y los microRNAs.**

**Investigador Principal:** Lucía Matute-Blanco^a^.

**Investigadores colaboradores:** David De Gonzalo-Calvo^b^, Juan Casanova-Sandoval^a^, Thalía Belmonte García^b^, Diego Fernández-Rodríguez^a^, Kristian Rivera^a^, Ignacio Barriuso^a^, Ferrán Barbé ^a,c^, Fernando Worner Diz ^a,b^.

**Filiaciones:**

Servei de Cardiologia. Hospital Universitari Arnau de Vilanova de Lleida^a^.

Grup TRRM. Institut de Recerca Biomèdica de Lleida (IRB LLEIDA). Hospital Universitari Arnau de Vilanova de Lleida^b^.

Servei de Pneumologia. Hospital Universitari Arnau de Vilanova de Lleida^c^.

**Dirección:** Avenida Rovira Roure 80. Lleida.

**Código Postal:** 25198.

**Teléfono:** 973-468024

**Índice**

1. Introducción
2. Antecedentes
   1. Evaluación fisiológica invasiva de la circulación coronaria.
      1. Reserva de Flujo Coronario: Coronary Flow Reserve (CFR).
      2. Reserva Fraccional del Flujo: Fractional Flow Reserve (FFR).
      3. Índice de Resistencia Microvascular: Index of Microvascular Resistance (IMR).
      4. Reserva Fraccional del Flujo: Fractional Flow Reserve (FFR).
      5. “Resting Full-Cycle Ratio” (RFR) y “Adjusted Resting Full-Cycle Ratio” (RFRa).
      6. Modelos computacionales angiográficos: Quantitative Flow Ratio (QFR) y Angiography-derived Index of Microcirculatory Resistance (IMRangio).
      7. Test de vaso-reactividad coronaria con acetilcolina.
   2. MicroRNAs y enfermedad cardiovascular
      1. Síntesis y función de los microRNAs.
      2. Potencial de los microRNAs como biomarcadores.
      3. Regulación de la expresión genética en la enfermedad cardiovascular: papel de los microRNAs.
      4. Relación entre los microRNAs y la enfermedad cardiovascular: resumen de la evidencia.
      5. Variabilidad de los microRNAs en los estudios publicados
3. Hipótesis
4. Objetivo principal
5. Objetivos secundarios
6. Material y métodos.
   1. Reclutamiento.
   2. Criterios de inclusión.
   3. Criterios de exclusión.
   4. Estimación tamaño muestral.
   5. Análisis estadístico
   6. Selección de microRNAs y otras muestras.
   7. Recogida, procesado, conservación y análisis de las muestras biológicas.
   8. Análisis bioinformático.
   9. Diseño.
   10. Coronariografía diagnóstica y evaluación fisiológica del árbol coronario.
   11. Determinación del patrón coronario afectado.
7. Interés científico de la investigación.
8. Análisis de la viabilidad del proyecto.
9. Bibliografía.
10. Anexos.

**INTRODUCCIÓN.**

El dolor torácico es uno de los principales motivos de consulta en cardiología, traduciendo principalmente la existencia de cardiopatía isquémica (1). No obstante, en una elevada proporción de pacientes con isquemia miocárdica, no se documentan lesiones coronarias obstructivas de las arterias coronarias epicárdicas en las pruebas invasivas (1,2).

En la circulación coronaria arterial se produce una progresiva ramificación de los vasos, constituyéndose dos compartimientos diferenciados que pueden comprometerse individualmente o de forma combinada: a) el “*compartimiento macrovascular”* constituido por las arterias epicárdicas (función de conducción); y b) el *“compartimiento microvascular”* constituido por las arteriolas (función regulatoria del flujo) y los capilares (función de intercambio). El progreso tecnológico ha posibilitado el desarrollo de nuevos índices coronarios fisiológicos invasivos que permiten un exhaustivo examen de ambos compartimientos arteriales, permitiendo discernir de forma precisa su afectación (2–5).

Por otro lado, los microRNAs (miRNAs) comprenden un abanico de pequeños fragmentos de RNA no codificante (nc-RNA) que influyen en la regulación post-transcripcional de la expresión génica; habiéndose demostrado que su expresión puede encontrarse alterada en la cardiopatía isquémica(6). Sin embargo, no existen trabajos de investigación que evalúen de forma integral el rol de los miRNAs en relación con el compartimiento coronario afectado(7).

Así, nuestro objetivo será evaluar prospectivamente, la relación de los diferentes patrones de afectación coronaria con los niveles de miRNAs circulantes.

**ANTECEDENTES**

**- Evaluación fisiológica invasiva de la circulación coronaria.**


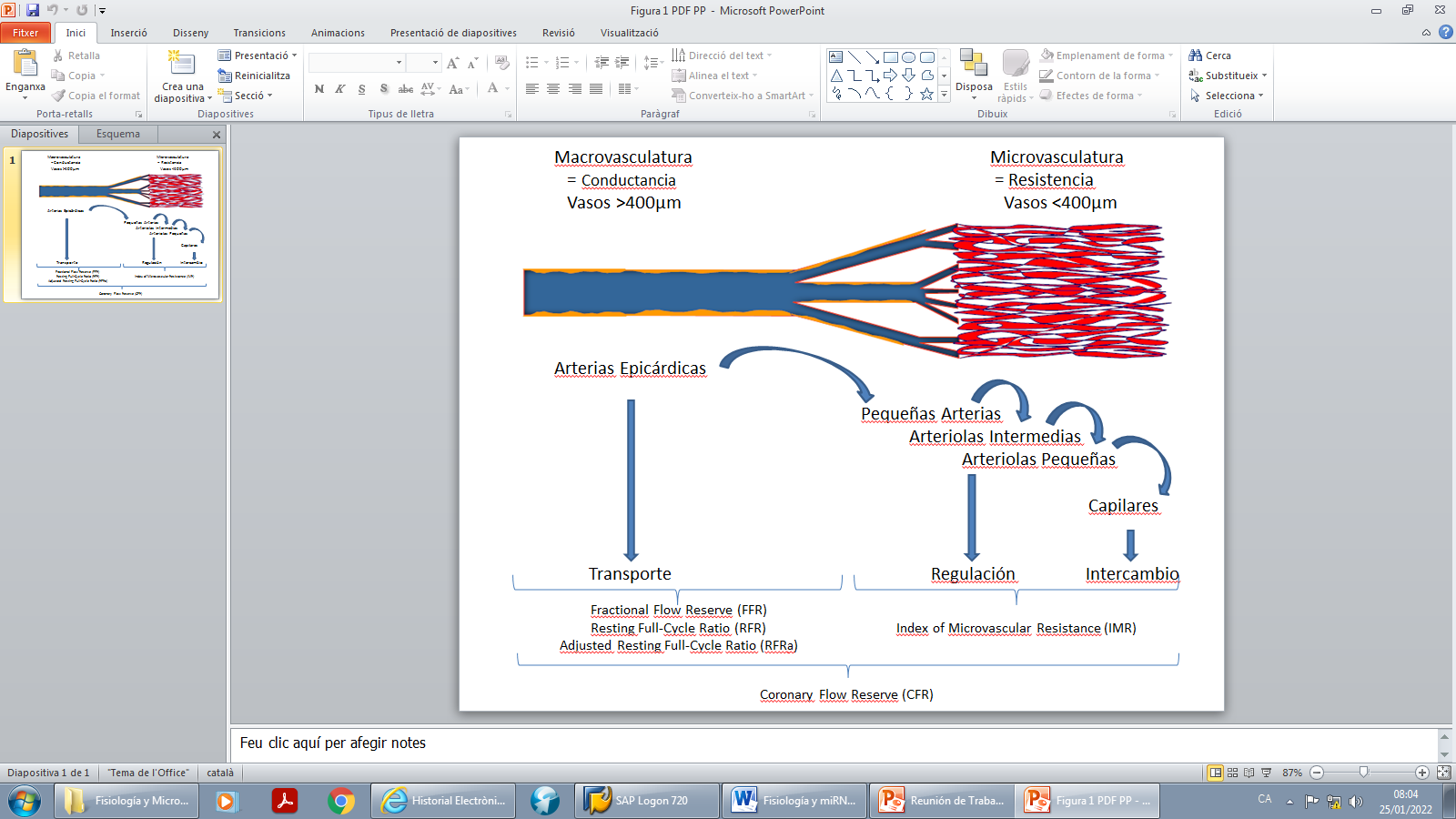
La circulación coronaria es el conjunto de vasos encargados de proveer de sangre rica en oxígeno y nutrientes al miocardio, siendo la función cardíaca altamente dependiente del mantenimiento y modulación del flujo coronario. El miocardio es uno de los tejidos con los requerimientos aeróbicos basales más altos del organismo (8-10 mL O2/min/100 g en el miocardio vs. 0.15 mL O2/min/100 g en el músculo esquelético) (3,8). A nivel anatómico la regulación del flujo coronario se estructura en dos compartimientos diferenciados. Por un lado, se encuentra el compartimiento macrovascular que comprende arterias > 400 micras (vasos de conductancia) cuya función es distribuir el flujo coronario a los distintos territorios, y por otro, el compartimiento microvascular que comprende arterias < 400 micras, arteriolas intermedias y pequeñas arteriolas (vasos de resistencia) cuya función es regular la cantidad de flujo coronario, mediante su vasodilatación o vasoconstricción, que llega a los capilares en los que se produce el intercambio de nutrientes y deshechos celulares (3,8).

Ilustración 1. Fisiología de la circulación coronaria

Los estudios hemodinámicos acerca de la circulación sanguínea coronaria se basan en la “Ley de Poiseuille” para circuitos hemodinámicos y su relación con la “Ley de Ohm” para los circuitos eléctricos. En base a ellas se establece que el caudal de fluido a través de un circuito se relaciona de forma proporcional con el gradiente de presión a través del mismo e inversamente proporcional a la resistencia del circuito(9).

| **Ley de Ohm** | | **Ley de Poiseuille** | |
| --- | --- | --- | --- |
| ΔVe  Ie =  Re | | ΔPcor ΔPcor  Qcor = =  RCor (8 μ L / π r^4^ ) | |
| Ie | Intensidad de Corriente | Qcor | Flujo Coronario |
| ΔVe | Gradiente de Voltaje Eléctrico | ΔPcor | Gradiente de Presión Transcoronario |
| Re | Resistencia Eléctrica | Rcor | Resistencia Coronaria |

Tabla 1. Equivalencia entre la ley de Ohm y la ley de Poiseuille

En condiciones fisiológicas basales la relación entre la presión y el flujo sanguíneo en las arterias coronarias no es lineal, observándose que en un amplio rango de presiones el flujo coronario se mantiene estable. Este fenómeno se conoce como “autorregulación coronaria”(8). No obstante, en situaciones de vasodilatación completa de los vasos de resistencia, ya sea inducida fisiológicamente mediante el ejercicio o farmacológicamente, por ejemplo, con adenosina, la relación entre el gradiente de presión y el flujo coronario se vuelve lineal permitiendo aplicar las consecuencias de la “Ley de Poiseuille” para obtención de índices fisiológicos coronarios y evaluar así la función circulatoria coronaria (8,10).

**- Reserva de Flujo Coronario: Coronary Flow Reserve (CFR)**(11)

El aumento de flujo desde la situación basal, donde rige el mecanismo de “autorregulación coronaria”, a la situación de hiperemia máxima, en la que el caudal de flujo coronario depende del gradiente de presión en la circulación coronaria; constituye un excelente indicador del estado funcional de la circulación, tanto a nivel macrovascular como microvascular. En condiciones normales, la circulación coronaria puede aumentar más de 5 veces en hiperemia máxima respecto a la situación basal.

Asimismo, el flujo coronario o “Coronary Flow” (CF) puede evaluarse mediante técnica de termodilución al determinar el “Tiempo de tránsito medio” o “Mean Time Transit” (Tmn) al inyectar suero salino a temperatura ambiente en la coronaria izquierda y cuantificar el cambio de temperatura de la “mezcla sangre-suero salino” entre 2 sensores de temperatura de una guía intracoronaria posicionada distalmente en la arteria arteria a evaluar. Así el CF sería la inversa del Tmn, y la CFR correspondería con razón entre el CF en hiperemia máxima y el CF en reposo, dando cuenta de la capacidad del lecho coronario para incrementar el CF en situaciones de altos requerimientos energéticos que requiriesen aumentar el gasto cardiaco y por ende el flujo coronario necesario para que el miocárdico pueda afrontar dicho aumento del gasto cardiaco. Se determina como sigue(11):

| **Coronary Flow (CF)** | |
| --- | --- |
| 1  CF =  Tmn | |
| **Coronary Flow Reserve (CFR)** | |
| Coronary Flow at Hyperemia 1 / Tmn Hyp Tmn Rest  CFR = = =  Coronary Flow at Rest 1 / Tmn Rest Tmn Hyp | |
| CF | Coronary Flow |
| Tmn | Mean Time Transit |
| CFR | Coronary Flow Reserve |
| Normal value of CFR | ≥ 2.0 |

Tabla 2. Coronary Flow (CF) y Coronary Flow Reserve (CFR)

**-** **Índice de Resistencia Microvascular: Index of Microvascular Resistance (IMR)**(12)**.**

El IMR es un índice desarrollado para evaluar la resistencia del compartimiento microvascular. Siguiendo la “Ley de Poiseuille”, combinada con el principio de generación de hiperemia máxima, se determina la presión en la distalidad del lecho macrovascular y el Tmn en hiperemia máxima.

En base a estos supuestos, se obtiene un índice coronario altamente relacionado con la disfunción microvascular de la siguiente forma:

| **Coronary Flow at Hyperemia** | | |
| --- | --- | --- |
| 1  Q Hyp =  Tmn Hyp | | |
| **Index of Microvascular Resistance (IMR)** | | |
| ΔPmicrov Hyp P at Distal LAD (Pd) – P Venous Sinus (Pv)  IMR = =  Q Hyp 1 / Tmn Hyp | | Dado que  Pv ≈ 0 |
| IMR = Pd × Tmn Hyp | | |
| Q Hyp | Coronary Flow at Hyperemia | |
| Tmn Hyp | Mean Time Transit at Hyperemia | |
| ΔPmicrov Hyp | Transmicrovascular Gradient at Hyperemia | |
| Normal value of IMR | < 25 | |

Tabla 3. Índice de resistencia microvascular.

**- Reserva Fraccional del Flujo: Fractional Flow Reserve (FFR)**(13,14)**.**

La FFR es un índice coronario hiperémico que permite realizar una evaluación funcional de las lesiones epicárdicas, basándose también en la relación lineal entre presión y flujo en el árbol coronario durante la hiperemia máxima.

En base a ello, el gradiente de presión translesional que genera una estenosis epicárdica es proporcional a la caída de presión a través de dicha lesión. Así, un valor de FFR de 0.60 para una lesión epicárdica se correspondería con una reducción del 40% del flujo coronario en hiperemia máxima (o reserva coronaria), atribuible a dicha lesión coronaria en comparación con la misma arteria si no presentara dicha estenosis. Es un índice con un amplio cuerpo de evidencia, tratándose del patrón-oro de los índices coronarios para la determinación de isquemia relacionada con enfermedad del compartimiento macrovascular. Se determina de la siguiente manera:

| **Fractional Flow Reserve (FFR)** | | |
| --- | --- | --- |
| At Hyperemia:  P distal to lesion (Pd) - P at Venous Sinus (Pv)  FFR =  P at Aorta (Pa) – P at Venous Sinus (Pv) | | Dado que  Pv ≈ 0 |
| At Hyperemia:  Pd  FFR =  Pa | | |
| Normal value of FFR | > 0.80 | |

Tabla 4. Reserva Fraccional del Flujo: Fractional Flow Reserve (FFR).

**- “Resting Full-Cycle Ratio” (RFR) y “Adjusted Resting Full-Cycle Ratio” (RFRa)**(15)**.**

Dada la necesidad de inducir hiperemia máxima para la valoración del FFR mediante fármacos vasodilatadores que pudiesen condicionar reacciones adversas y/o que dificultasen la generalización de las técnicas de evaluación fisiológica de las lesiones epicárdicas, se desarrollaron índices coronarios de reposo o no hiperémicos para la evaluación del compartimiento macrovascular. Se trata de índices que no requieren la administración de drogas y presentan una elevada correlación con los valores de FFR.

Entre estos índices destaca el RFR. Se trata de un índice que sin necesidad de vasodilatadores permite valorar la significación hemodinámica de las lesiones epicárdicas, identificando el menor valor del cociente presión distal a la estenosis (Pd) / presión aórtica (Pa) durante todo el ciclo cardiaco en reposo y, en oposición a otros índices de reposo, sus mediciones serían menos dependientes de la morfología de las ondas de presión, la señal eléctrica y las variaciones fásicas en la resistencia microcirculatoria.

Sin embargo, y aunque la correlación entre RFR y FFR es buena, un porcentaje no despreciable de lesiones pueden ser mal clasificadas tomando el FFR como referencia. Por ello, en nuestro grupo hemos desarrollado el índice RFRa que permite ajustar las diferencias en base a otros parámetros clínicos y/o angiográficos, aumentado así la concordancia entre las técnicas.

| **Resting Full-Cycle Ratio (RFR)** | |
| --- | --- |
| Pd  FFR = Lowest value of during whole cardiac cycle  Pa | |
| **Adjusted Resting Full-Cycle Ratio (RFRa)** | |
| RFRa = 0.009 + 0.912*RFR + 0.023*CKD - 0.019*Non-LAD - 0.017*ACS – 0.005*IHD | |
| CKD | Chronic kidney disease |
| Non-LAD | Non left anterior descending artery lesions |
| ACS | Acute coronary syndrome |
| IHD | Prior ischemic heart disease |
| Normal value of RFR | > 0.89 |
| Normal value of RFRa | > 0.8172 |

Tabla 5. Resting Full-Cycle Ratio. Adjusted Resting Full-Cycle Ratio.

**- Modelos computacionales angiográficos para la evaluación de los compartimientos coronarios**(16)**.**

# Basándose en los principios anteriormente expuestos y en la dinámica computacional de flujos, se han desarrollado modelos computacionales que permiten estimar la afectación de los compartimientos coronarios mediante índices coronarios basados en la angiografía. El “Quantitative Flow Ratio” (QFR) es un índice que ha mostrado presentar una buena correlación con el FFR, siendo superior a la angiografía convencional en la evaluación de la potencial isquemia generada por las lesiones coronarias epicárdicas. Además, más recientemente se han desarrollado incluso índices coronarios basados en la angiografía que permiten estimar la resistencia microvascular sin necesidad de emplear guías de presión como el “Angiography-derived Index of Microcirculatory Resistance” (IMRangio).

**- Test de vaso-reactividad coronaria con Acetilcolina**(1)**.**

En condiciones normales, una arteria provista de un endotelio sano responde a la acetilcolina con liberación de óxido nítrico, generando vasodilatación. En casos de bloqueo de la acción de la enzima óxido nítrico sintasa o denudando la pared arterial del endotelio, se genera por el contrario vasoconstricción, secundaria a una estimulación de los receptores muscarínicos del músculo liso, no contrarrestada por el óxido nítrico de origen endotelial. Así, la infusión de acetilcolina puede emplearse para evaluar la función endotelial y la adecuada tonicidad de los vasos coronarios. Un porcentaje relevante de pacientes con angina sin enfermedad coronaria obstructiva asociada pueden presentar este tipo de alteraciones. Aunque se han empleado distintos métodos para desenmascarar esta entidad, actualmente el más empleado es el test de infusión intracoronaria de acetilcolina. Su objetivo para determinar la presencia de disfunción endotelial o alteraciones tónicas se basa en la reproducción de los síntomas, el desencadenamiento de vasoconstricción y la detección de alteraciones electrocardiográficas. A continuación, se detallan la evaluación de los resultados de dicha prueba:

| **Test de Infusión de Acetilcolina Intracoronario: Criterios Diagnósticos** | |
| --- | --- |
| • Negativo con respuesta vasodilatadora (normal):  -No clínica  -No cambios ECG  -Vasodilatación tras Ach (respecto basal) | • Negativo con respuesta vasoconstrictora:  -No clínica  -No cambios ECG  -Sin constricción ≥ 90% (respecto NTG)  -Leve vasoconstricción (respecto basal) |
| • Vasoespasmo epicárdico:  -Reproducción de síntomas  -Ascenso o Descenso ST  -Constricción ≥ 90% (respecto NTG) | • Vasoespasmo microvascular:  -Reproducción de síntomas  -Descenso ST  -Sin constricción ≥ 90% (respecto NTG) |

Tabla 6. Test de vaso-reactividad coronaria con acetilcolina.

**- MicroRNAs y enfermedad cardiovascular**

**- Síntesis y función de los microRNAs.**

En el genoma humano, los genes que codifican proteínas representan únicamente el 1.1%, mientras que más del 70% del genoma se transcribe dando lugar a RNA no codificante (nc-RNA) que desempeña importantes funciones en la regulación de procesos fisiopatológicos y en la homeostasis celular.

Los miRNAs son un tipo de pequeños nc-RNA (17 a 25 nucleótidos) que juegan un importante papel en la regulación de la expresión génica. Hasta la fecha, los miRNAs son los nc-RNA más estudiados y descritos en la literatura (17).

El concepto inicial de que los miRNAs regulan la expresión de los genes se desarrolló hace casi 30 años, en 1993 en los laboratorios de Ambros y Ruvkun al estudiar el desarrollo del nemátodo *Caenorhabditis elegans* (18). Varios estudios han demostrado que los miRNA se conservan evolutivamente en todas las especies y suelen expresarse de forma ubicua (19) (20)(21)

Los miRNAs pueden regular negativamente la expresión génica a nivel postranscripcional uniéndose al RNA mensajero (mRNA) y provocando el silenciamiento génico mediante la inhibición de la traducción y/o degradación del mRNA; así como activar la transcripción de determinados genes diana. Se ha estimado que los miRNAs controlan la actividad del 30-50% de los genes que codifican proteínas, jugando un importante papel en la regulación de varios procesos biológicos, incluida la embriogénesis, la proliferación y la diferenciación celular, la apoptosis o tumorogénesis (22).

En el sistema cardiovascular, los miRNAs regulan diversos procesos que incluyen el crecimiento y la contractilidad de los cardiomiocitos, el desarrollo y mantenimiento del ritmo cardíaco, la formación de placa, el metabolismo de los lípidos y la angiogénesis (6). La expresión alterada de determinados miRNAs puede encontrarse en muestras sanguíneas de pacientes con diversas enfermedades cardiovasculares, lo que los convierte en atractivos candidatos como biomarcadores.

**- Potencial de los microRNAs como biomarcadores.**

Aunque los miRNAs se describieron inicialmente como reguladores intracelulares, se han detectado en fluidos corporales de forma estable. Los microARN extracelulares participan en la comunicación intercelular a nivel autócrino, paracrino y endocrino y han estado implicados tanto en respuestas fisiológicas como adaptativas, así como en la aparición y desarrollo de estados de enfermedad. Así, la concentración y la composición extracelular de miRNA reflejan estados fisiopatológicos y ofrecen información sobre el fenotipo molecular del paciente en diferentes condiciones (23–26).

Tal y como ha revisado nuestro grupo, los miRNAs extracelulares tienen las propiedades bioquímicas óptimas para convertirse en excelentes biomarcadores: a) pueden obtenerse mediante técnicas mínimamente invasivas en muestras clínicas; b) son altamente estables y tienen una larga semivida dentro de la muestra; c) pueden cuantificarse mediante técnicas estándar ya disponibles en los laboratorios clínicos: PCR de transcripción inversa cuantitativa (qPCR); d) los perfiles globales podrían obtenerse en un solo experimento mediante RTqPCR, o técnicas relativamente accesibles como la secuenciación de nueva generación o microarrays(27). A partir de estas características, el perfil de miRNA circulante constituye una nueva generación de biomarcadores en diversas enfermedades cardiovasculares.

**- Relación entre los microRNAs y la enfermedad cardiovascular: resumen de la evidencia.**

Actualmente ya son muchos los miRNAs relacionados, a través de diferentes mecanismos fisiopatológicos, con la enfermedad cardiovascular. A continuación, resumimos la evidencia actual acerca de los principales miRNAs evaluados en los escenarios clínicos relacionados con el objeto de nuestra investigación; a saber, la aterosclerosis y la enfermedad arterial coronaria.

| **miRNAs y aterosclerosis** | |
| --- | --- |
| miR-126-3p | Uno de los primeros miRNAs identificados en el desarrollo de ateroesclerosis.  Se expresa altamente en células endoteliales y su expresión es esencial en el desarrollo vascular.  Niveles reducidos favorece la formación de placa.  Inhibe la expresión de VCAM-1, molécula expresada por las células endoteliales que permite la adhesión y migración de los leucocitos a la pared vascular. (28) |
| miR-21-5p | Relacionado con estadios precoces del proceso ateroesclerótico en pacientes con hipertensión arterial(29). |
| miR-155-5p | Expresión inducida por TNF-α e IFN-β. Aumenta la población de granulocitos y monocitos durante la inflamación. Regula también expresión del gen AT1R (receptor de angiotensina II tipo 1) encontrándose asociado a la regulación de la presión arterial y la ateroesclerosis(30). |
| miR-143-5p, miR-145-5p | Se expresan en las células del músculo liso vascular (VSMC) e inhiben la migración y proliferación dentro de la placa ateroesclerótica en desarrollo. (31) |
| miR-133a-5p | Inhibe el crecimiento y proliferación de las células del músculo liso vascular. (32) |
| miR-122 -5p | Favorece la ateroesclerosis a través de la regulación de la homeostasis del colesterol. También favorece la inflamación y fibrosis miocárdica. (33) |
| miR-100-5p | Suprime la expresión de moléculas de adhesión endotelial atenuando la interacción entre el endotelio y los leucocitos y reduciendo la inflamación de las placas ateromatosas. (34) |
| **miRNAs y Enfermedad Arterial Coronaria** | |
| miR-1-3p, miR-133a-3p, miR-208a-3p, miR-499a-5p | Presentan sobreexpresión en pacientes con síndrome coronario agudo y se relacionan con la extensión del daño y pronóstico de los pacientes(35,36). |
| miR-126-3p, miR-17-5p, miR-92a-3p | Infra expresión de estos miRNA en pacientes con enfermedad arterial coronaria estable en comparación con sujetos sanos(37). |
| miR-126-3p | Ha demostrado presentar asociación positiva con IAM en población general en un seguimiento de 10a (estudio prospectivo). (38) |
| miR-197-3p, miR-223-3p | Ha demostrado presentar asociación positiva con IAM en población general. (39) |
| miR-126-3p, miR-145-5p | La presencia de gradientes transcoronarios se relaciona con la presencia de placas ateromatosas inestables. (40) |
| miR-1-3p | Inhibe la expresión del gen Spred1 y promueve la angiogénesis y la formación de circulación colateral. En relación con la cardiogénesis y la regulación de la diferenciación de las células madre en cardiomiocitos. (41,42) |
| miR-214-3p | Sus niveles presentan correlación con la expresión de factor de crecimiento endotelial vascular. Un aumento de la concentración de este RNA puede ser usado para predecir la presencia y severidad de las lesiones coronarias en pacientes en enfermedad arterial coronaria. (43) |
| miR-499a-5p | Altamente enriquecido en el corazón embrionario  • Involucrado en las etapas cardiogénicas tardías  • Responsable de la diferenciación terminal de mioblastos a cardiomiocitos y especificación de fibra muscular rápida / lenta (44,45) |
| miR-208a-3p | Involucrado en las últimas etapas del desarrollo cardíaco  • Relacionado con el compromiso de cardiomiocitos de mioblastos  • Regular la expresión de la cadena pesada de miosina cardíaca (proteína contráctil principal) (44) |
| miR-142 | Potencial marcador para predecir MACE en pacientes con enfermedad arterial coronaria después de intervencionismo coronario percutáneo.(46) |

Tabla 7. Principales microRNAs asociados a la enfermedad cardiovascular (ECV) y su forma de actuación.

**- Variabilidad de los microRNAs en los estudios publicados.**

Como referimos previamente, la evidencia acerca de los miRNAs ha aumentado de forma muy importante en los últimos años. Sin embargo, es importante destacar que la utilidad de los miRNAs como biomarcadores puede variar entre los distintos estudios. Factores como el escenario clínico, el tipo de muestra, así como el momento de obtención y su procesado pueden afectar a los resultados reportados en los distintos estudios (42-48). En un reciente metaanálisis donde se analizan y comparan los miRNAs aislados en pacientes con enfermedad arterial coronaria respecto a sujetos sanos muestra sobreexpresión de determinados miRNAs en los pacientes con enfermedad coronaria; mientras que otros miRNAs mostraban resultados incongruentes con sobreexpresión en algunos estudios e infraexpresión en otros (47). Este estudio también muestra interesantes comparaciones entre los miRNAs aislados en enfermedad coronaria estable y en síndrome coronario agudo comparado con controles sanos como muestra la siguiente tabla.

| Enfermedad coronaria (cualquier tipo) vs controles sanos. |
| --- |
| Sobreexpresión en enfermedad coronaria: miR-1-3p, miR-499a-5p, miR-133a-3p, miR-208a-3p/208b-3p, miR-21-5p, miR-142-5p, miR-145-5p |
| Sobreexpresados e infraexpresados: miR-126-3p, miR-208b-5p, miR-92a-3p, miR-145-5p y miR-142-5p |
| Enfermedad coronaria estable vs controles sanos. |
| MiR sobreexpresados: miR-125a-5p, miR-187-3p, miR 502-5p |
| MiR infraexpresados: miR-145-5p, miR-29b-3p, let-7 |
| Sobre e infraexpresados: miR-126-3p, miR-155-5p, miR-17-5p, miR-92a-3p |
| Síndrome coronario agudo vs controles sanos. |
| MiR sobreexpresados: miR-1-3p, miR-499a-5p, miR-208a-3p/208b-3p, miR-133a-3p, miR-133b-3p, miR-27a-3p, miR-30e-5p, miR-93-5p, miR-21-5p |
| Sobre e infraexpresados: let-7, miR-208b-3p, miR-126-3p, miR-145-5p, miR-134, miR-223-3p, miR-142-5p |
| Síndrome coronario agudo vs enfermedad coronaria estable. |
| Sobreexpresados en síndrome coronario agudo: miR-21, miR-208a-3p/208b-3p, miR-133a-3p, miR-133b-3p, miR-140-5p, miR-146a-5p, miR-499a-5p, miR-27a-3p, miR-27b-3p, miR-451a, miR-29a-3p, miR-29c-3p |
| Sobreexpresados e infraexpresados: miR-30a-5p, let-7, miR-142-5p, miR26a-5p, miR26b-5p, miR-92a-3p, miR-150-5p, miR-22-3p, miR-223-3p |

Tabla 8. MicroRNAs identificados en pacientes con enfermedad arterial coronaria (estable y/o síndrome coronario agudo). En verde microRNAs con infraexpresión. En rojo microRNAs con sobreexpresión. En amarillo microRNAs con sobre/infraexpresión. (47)

Es por ello que, a pesar del importante volumen de literatura sobre los miRNAs en diferentes cardiopatías, por lo que hay que ser cautelosos a la hora de interpretar los datos ofrecidos por los distintos estudios y emplear una metodología robusta que permita una rigurosa evaluación de los miRNAs en cualquier proyecto de investigación que contemple la evaluación de estos biomarcadores.

**HIPÓTESIS**

Los distintos patrones de afectación coronaria se asocian con la sobreexpresión o infraexpresión de determinados miRNAs.

**OBJETIVO PRINCIPAL**

Caracterizar la expresión de los miRNAs en función del compartimiento coronario afectado en pacientes con dolor torácico.

**OBJETIVOS SECUNDARIOS**

1.- Evaluar la asociación de la expresión de los miRNAs con el grado de estenosis y la extensión de la afectación de las arterias coronarias epicárdicas.

2.- Evaluar la asociación de los niveles de miRNAs con índices basados en la angiografía para determinación de los compartimientos coronarios.

3.- Evaluar la asociación de la expresión de los miRNAs con la presencia de factores de riesgo cardiovascular y/o enfermedad vascular establecida.

4.- Evaluar la asociación de la expresión de los miRNAs con otros marcadores hematológicos y bioquímicos.

**MATERIAL Y MÉTODOS.**

**- Reclutamiento.**

**S**e incluirán pacientes con angina remitidos para realización de coronariografía, del Hospital Universitari Arnau de Vilanova de Lleida. Tras confirmar que los pacientes sean elegibles para el estudio y de que hayan firmado el consentimiento informado (**Anexo 1**), los pacientes serán incluidos en el estudio.

**- Criterios de inclusión:**

1. Edad ≥ 18 años.
2. Pacientes con dolor torácico sugestivo de angina evaluados por un cardiólogo remitidos para coronariografía diagnóstica y eventual angioplastia coronaria.
3. Ecocardiograma que descarte causas cardiacas no coronarias de dolor torácico.
4. Consentimiento informado.

**- Criterios de exclusión:**

1. Alergia al contraste no susceptible de recibir pre-medicación.
2. Asma bronquial severa o intolerancia a la adenosina.
3. Bloqueo auriculoventricular (≥ 2º grado) o intolerancia a la acetilcolina.
4. Infarto agudo de miocardio con elevación del segmento ST.
5. Infarto agudo de miocardio sin elevación del segmento ST.
6. Shock cardiogénico.
7. Oclusión total de cualquier arteria coronaria que impida la medición con guías de presión-temperatura.
8. By-pass coronario previo.
9. Mujeres con posibilidad de estar embarazadas.
10. Disfunción renal con un filtrado glomerular estimado < 30 mL/min/1.73m2.
11. Incapacidad para comprender la naturaleza del estudio y/o firmar el consentimiento informado.
12. Cualquier otra condición médica que en opinión del investigador pueda comportar problemas de seguridad para los pacientes o puedan alterar los resultados del estudio.

*NOTA: *Dado que, en la práctica clínica habitual, una proporción importante de pacientes son remitidos para coronariografía sin prueba de detección de isquemia positiva previa e incluso con prueba de detección de isquemia negativa ante una sospecha elevada de dolor anginoso, en nuestro estudio no será obligatorio la presencia de prueba de detección de isquemia para ser incluido en el estudio quedando a criterio del cardiólogo emisor solicitar la coronariografía.*

**- Estimación del tamaño muestral.**

En base a estudios previos de nuestro grupo y estudios previos para la determinación de miRNAs en presencia de disfunción endotelial coronaria han reclutado en torno a 40-50 pacientes para completar la investigación (7,48–50). En nuestro caso, se estudiará la relación entre los miRNAs con los distintos patrones de afectación en función del compartimiento coronario afectado, a saber: Grupo 1 (Angina Macrovascular y Microvascular); Grupo 2 (Angina Macrovascular); Grupo 3 (Angina Microvascular); y Grupo 4 (Dolor Torácico No Coronario). Dado que el Grupo 4 corresponde a pacientes sin alteraciones de la circulación coronaria, se utilizará como grupo de control respecto a los otros grupos hasta completar un reclutamiento de 25 pacientes por grupo, en cuantía similar a otros grupos control en estudios similares, totalizando 100 pacientes. Asimismo, ante la posibilidad de que algunos pacientes presenten espasmo macrovascular y/o microvascular que podría solaparse con alguno de los otros grupos, se ha definido el Grupo 5 (Angina Espástica Macrovascular o Microvascular) que no computará a efectos de estimación del tamaño muestral.

**- Análisis Estadístico.**

Las variables continuas serán expresadas como media ± desviación estándar (DE) o mediana con rango intercuartílico (RCI), de acuerdo con su distribución. Según sea apropiado, se empleará el test U de Mann-Whitney para comparar variables continuas, mientras que se empleará el test de Chi-cuadrado o el test de Fisher para comparar las variables categóricas. En caso de requerirse estudios de subgrupos dentro de cada grupo (ejemplo: pacientes del grupo 1 [angina macrovascular y angina microvascular] y diabetes), se realizará la prueba de ANOVA Factorial cuando se supongan varianzas iguales o la prueba de Brown Forsythe cuando se supongan varianzas desiguales, seguido de un análisis posthoc con la corrección de Bonferroni para comparaciones múltiples. Los resultados se mostrarán como media con un intervalo de confianza (IC) del 95%. La igualdad de varianzas entre grupos se calculará para cada variable dependiente mediante la prueba de leven, con la corrección de Bonferroni para probar la homogeneidad de la varianza entre todos los niveles de comparación.

Se empleará un nivel alfa de 0.05 para evaluar la significación estadística. Los datos se analizarán utilizando el software SPSS o mediante STATA.

**- Selección de microRNAs y otras muestras.**

De forma imprescindible se determinarán aquellos miRNAs que hayan mostrado únicamente sobre o infraexpresión en estudios previos de aterosclerosis coronaria o enfermedad coronaria estable, a saber:

- Sobreexpresión: miR-1-3p, miR-21-5p, miR-133a-3p, miR-133b-3p, miR-208a-3p,

miR-208b-3p, miR-125a-5p, miR-187-3p, miR-499a-5p, miR-502-5p.

- Infraexpresión: miR-100-5p, miR-143-3p, miR-145-3p, miR-29b-5p.

Asimismo, dado que los kits para la determinación de miRNAs permiten el análisis de múltiples miRNAs en cada muestra, se determinarán de acuerdo con los expertos en biología molecular otros potenciales candidatos. Asimismo, se extraerán muestras que corresponden a la práctica habitual como hemograma, bioquímica, lípidos…

**- Recogida, procesado, conservación y análisis de las muestras biológicas.**

En cada paciente, previo a la coronariografía y a la administración cualquier fármaco necesario para la realización de la misma, especialmente la heparina requerida para los procedimientos coronarios, así como de contraste yodado; se realizará una extracción de 6 mL de sangre (tubo EDTA K2). La recolección y preparación de muestras de plasma se realizará de acuerdo con los procedimientos operativos estandarizados del NCI (EEUU). Las muestras serán almacenadas en el Biobanco del IRBLleida perteneciente a la Plataforma Nacional de Biobancos. Los análisis específicos de miRNAs se realizarán en el Laboratorio del grupo TRRM del Institut de Recerca Biomèdica de Lleida. El análisis del resto de parámetros bioquímicos y/o hematológicos se realizará en los laboratorios del Hospital Universitari Arnau de Vilanova de Lleida. En caso de no observar diferencias entre los miRNA en los distintos grupos de estudios las muestras se conservarán en el Biobanco del IRBLleida de cara a valoración de otros biomarcadores en los que el grupo TRRM tiene experiencia, como son lncRNAs, circRNAs, proteínas.

El aislamiento de RNA y la cuantificación de miRNA se realizará por personal experimentado sin acceso a los datos clínicos. Todos los experimentos se realizarán utilizando la técnica *gold-standard*; RT-qPCR, en condiciones estandarizadas en el mismo laboratorio y de acuerdo con la metodología previa utilizada por el grupo de investigación(50–52). Brevemente, el RNA total se aislará de 200 μL de muestras de plasma congeladas utilizando el kit miRNeasy Serum/Plasma Advanced (Qiagen), de acuerdo con las instrucciones del fabricante. El RNA sintético de *Caenorhabditis elegans* miR-39-3p (cel-miR-39-3p) se agregará como RNA de referencia externo. La purificación de RNA se realizará con las columnas de centrifugación RNeasy UCP MinElute de acuerdo con las recomendaciones del fabricante. El RNA se almacenará en un congelador a -80 °C hasta su posterior análisis. El análisis de RT-qPCR se realizará de acuerdo con las recomendaciones de las guías MIQE(53). La cuantificación de miRNA se realizará de acuerdo con el protocolo del sistema miRCURY® LNA® RT Kit (Qiagen), que ofrece una precisión y reproducibilidad óptimas(54). Las reacciones de RT se realizarán utilizando el kit miRCURY LNA RT (Qiagen). El cDNA se almacenará a -20°C. La PCR cuantitativa (qPCR) se realizará con el kit miRCURY LNA SYBR® Green PCR (Qiagen) utilizando 384-well miRCURY LNA miRNA Custom PCR Panels (Qiagen). La qPCR se realizará en un sistema de PCR en tiempo real QuantStudio ™ 7 Flex (Thermo), seguido de un análisis de *melting curve*. Las curvas de amplificación de qPCR se evaluarán con el software QuantStudio Software v1.3 (Thermo). Un Cq superior a 35 ciclos se considerará indetectable y se censurará al nivel mínimo observado para cada miRNA. Los miRNAs en los que el 80% de las muestras cumplen estos criterios se considerarán por debajo del límite de detección. La cuantificación relativa se realizará mediante el método 2^− dCq^, donde:

| ΔCq = Cq_miRNA_ − Cq_cel-miR-39-3p_. |
| --- |

**- Análisis bioinformático.**

Se realizará conforme a publicaciones previas del grupo de investigación(25) . Las rutas moleculares afectadas por los de transcritos identificados se analizarán mediante la herramienta de computación web DIANA-miRPath v3.0 que combina estimaciones y datos experimentales con la herramienta KEGG para identificar rutas moleculares.

**- Diseño**

Ilustración 2. Diagrama explicativo de la Fase Clínica.

**- Coronariografía diagnóstica y evaluación fisiológica del árbol coronario.**

Tras la realización de la angiografía coronaria diagnóstica se realizará un análisis estandarizado de las lesiones coronarias en cuanto a porcentaje de estenosis y longitud de la lesión mediante “quantitative coronary angiography” (QCA), al objeto de evitar la variabilidad en la estimación visual de las lesiones coronarias. Posteriormente, se realizará una valoración funcional de las lesiones epicárdicas con guía de presión, primero con la medición de los índices coronarios no hiperémicos: “Resting full-cycle ratio” (RFR) y RFR ajustado por los predictores de discordancia (“Adjusted RFR), y a continuación mediante los índices coronarios hiperémicos “Fractional Flow reserve” (FFR), “Coronary Flow Reserve” (CFR) e “Index of Microcirculatory Resistance” (IMR) posicionando la guía de presión en el segmento distal de cada arteria a evaluar, pudiendo interrogarse más de una arteria en un mismo paciente. Asimismo, en los pacientes remitidos para coronariografía que posean test de isquemia positivo previo cuyas arterias epicárdicas no se consideren tributarias de evaluación, se posicionará la guía de presión también en la arteria que corresponda al territorio compatible con la isquemia detectada en el test. En caso de lesiones coronarias no susceptibles de evaluación fisiológica, se posicionará la guía de presión en el segmento distal de la arteria descendente anterior, evaluándose el “Coronary Flow Reserve” (CFR) y el “Index of Microcirculatory Resistance” (IMR) en el territorio de la descendente anterior. Las medidas serán obtenidas mediante el dispositivo ″PressureWire™ X Guidewire de 0.014” (Abbott Vascular Inc., Santa Clara, CA,). Para la inducción de hiperemia se empleará adenosina como fármaco vasodilatador. También, se evaluarán para cada lesión el índice derivado de la angiografía “Quantitative Flow Ratio” (QFR) y para cada arteria el “Angiography-derived Index of Microcirculatory Resistance” (IMRangio).

En cuanto a la realización del test de vaso-reactividad coronaria se empleará la acetilcolina según lo previamente expuesto. Dado el riesgo de espasmo epicárdico en caso de isquemia macrovascular derivada de lesiones epicárdicas, se realizará el test de acetilcolina solo en pacientes que no presenten valores patológicos de FFR (> 0.80). En el **Anexo 2** se presentan más detalladamente las recomendaciones para la realización de los estudios de fisiología coronaria.

**- Determinación del patrón coronario afectado.**

Una vez obtenidos los parámetros, se determinará el patrón de angina correspondiente a cada paciente, en función de los valores de FFR, CFR e IMR, realizándose el tratamiento correspondiente a los hallazgos obtenidos. La decisión de tratamiento médico, percutáneo o quirúrgico de las lesiones coronarias epicárdicas que condicionen isquemia se realizará en base al FFR.

La afectación macrovascular se definirá cuando el valor de FFR sea ≤ 0.80 y la afectación microvascular cuando el valor de IMR sea ≥ 25 o el CFR sea < 2.0 en presencia de un FFR > 0.80. Los patrones de angina dependerán de las distintas combinaciones según la afectación de los compartimientos coronarios, a saber: Grupo 1 (Angina Macrovascular y Microvascular); Grupo 2 (Angina Macrovascular); Grupo 3 (Angina Microvascular); y Grupo 4 (Dolor Torácico No Coronario). Dado que el presente protocolo también contempla una aproximación al estudio del espasmo coronario se considerará un grupo adicional, mediante la valoración del test de acetilcolina en pacientes que presenten angina microvascular o dolor torácico no coronario (Grupos 3 y 4). Los pacientes que presenten espasmo macro o microvascular en dicho test serán encuadrados en el Grupo 5 (Angina Espástica Macro o Microvascular).

**INTERÉS CIENTÍFICO DE LA INVESTIGACIÓN.**

La presente propuesta de investigación podría aportar resultados relevantes acerca de la caracterización epigenómica de la angina de pecho, mediante la determinación de miRNAs.

Tradicionalmente, se entiende la enfermedad arterial coronaria como un fenómeno que comienza por la disfunción endotelial y, a medida que progresa, afecta progresivamente a todo el árbol coronario (55). Asimismo, se considera que este proceso se dilata por un largo periodo de tiempo, lo que conlleva que los pacientes con enfermedad arterial coronaria puedan presentar una fase preclínica muy prolongada hasta el desarrollo de isquemia miocárdica. Sin embargo, una vez alcanzada esta fase de la enfermedad, uno de los principales factores pronósticos es la presencia de isquemia. Es de resaltar también la heterogeneidad de la presentación de la cardiopatía isquémica, existiendo datos que muestran que en los pacientes puede existir afectación microvascular o macrovascular aisladas, así como una combinación de estas (56).

Hasta la fecha, únicamente un reciente trabajo español (57) que muestra la utilidad de los miRNAs para distinguir la miocarditis aguda del síndrome coronario agudo ha despertado verdaderamente el interés de los cardiólogos clínicos acerca de estos marcadores de enfermedad tan prometedores en otro tipo de patologías. En relación con la enfermedad arterial coronaria, salvo el citado trabajo, la relevancia clínica de los miRNAs continúa siendo escasa. Ello puede ser debido a que el hallazgo de nuevos marcadores, que aporten información clínica adicional con respecto a la aportada por los factores clínicos, bioquímicos o de diagnóstico por la imagen, es dificultosa dado el gran desarrollo de la investigación en dichos campos desde hace varias décadas, especialmente en los pacientes que sobreviven a un infarto agudo de miocardio.

En nuestra opinión, la gran fortaleza a priori de esta investigación radicaría en la determinación robusta de 4 grupos de comparación, con las técnicas más específicas para su caracterización (estudios de fisiología coronaria), que contemplarían las tres posibles combinaciones de afectación de los compartimientos del árbol coronario (Grupos 1 a 3) y un grupo de pacientes sin cardiopatía isquémica (Grupo 4). Asimismo, un último grupo (Grupo 5), aportaría información adicional sobre la presencia de espasmo coronario. La definición precisa de estos grupos permitiría valorar de forma robusta la sobreexpresión o infraexpresión de los miRNAs, e incluso valorar la existencia de gradientes en los miRNAs en función de la extensión de la isquemia miocárdica y de la cantidad de compartimientos vasculares coronarias afectados.

Hasta ahora, escasos trabajos han valorado los miRNAs en forma similar a nuestra propuesta de investigación, como en la disfunción endotelial coronaria (7). Sin embargo, hasta donde conocemos no se ha evaluado específicamente el papel de los miRNAs en relación con índices de disfunción microvascular como el IMR ni se ha desarrollado un estudio que intente comprender globalmente el papel de los mismos en función de los compartimientos vasculares coronarios involucrados en la presencia de isquemia miocárdica.

También es reseñable, que la investigación en fisiología coronaria está experimentado un nuevo auge, y en nuestra opinión, las aportaciones en este campo serían muy valiosas. Además, la determinación de patrones de miRNAs podría redundar en una oportunidad para mejorar el tratamiento de los pacientes e incluso en la reducción de la necesidad de realización de pruebas fisiológicas invasivas. Por todo ello, consideramos que el presente estudio podría ser de gran interés científico.

Además, se establecerá un plan de contingencia en caso de no observar diferencias entre los miRNAs en los distintos grupos de estudios, puesto que contaremos con un banco de muestras que permitiría analizar otros biomarcadores donde el grupo de investigación posee experiencia. Asimismo, se establecerá un plan de difusión dirigido al público en general y se realizarán acciones para informar a los segmentos de población de especial interés/vulnerabilidad en el ámbito del estudio. La información obtenida se pondrá a disposición del mundo científico y del público en general en colaboración con el Departamento de Comunicación del IRBLleida. El IRBLleida ha dado varios pasos para la difusión de mensajes científicos de una forma clara y comprensible. Utilizaremos el sitio web del IRBLleida (https://www.irblleida.org/es/) que contiene información de proyectos, publicaciones y grupos de investigación. Para realizar la investigación más accesible a la sociedad, utilizaremos plataformas de comunicación digital. Asimismo, algunos de los investigadores son miembros del programa Europeo COST CardioRNA (https://cardiorna.eu/). Uno de los grupos de trabajo de este programa está centrado puramente en la comunicación de los resultados obtenidos.

**ANÁLISIS DE LA VIABILIDAD DEL PROYECTO.**

Consideramos que el proyecto es viable por los siguientes motivos. La Unidad de Hemodinámica del Hospital Universitari Arnau de Vilanova de Lleida realiza más de 1.110 coronariografía diagnósticas y más de 500 angioplastias coronarias al año y por consiguiente la realización de los estudios de fisiología coronaria invasiva podría llevarse a cabo en un periodo temporal razonable.

Asimismo, el Institut de Recerca Biomèdica de Lleida cuenta con la tecnología necesaria para realizar el procesamiento, conservación de las muestras y análisis de miRNAs, con amplia experiencia y publicaciones en dicho campo. Además, en el Servicio de Cardiología del Hospital Universitari Arnau de Vilanova de Lleida contamos con Unidad de Investigación con enfermería específicamente destinada a este cometido lo que asegura la extracción protocolizada de las muestras.

También es de destacar que el Servicio de Cardiología Hospital Universitari Arnau de Vilanova tiene experiencia en este ámbito, como muestra el hecho de que ha realizado investigaciones acerca de la validación y desarrollo de índices coronarios no hiperémicos(58), así como de patologías que presentan disfunción microvascular, como el síndrome de TakoTsubo (59).

**BIBLIOGRAFÍA.**

1. Knuuti J, Winjs W, Saraste A, Capodanno D, Barbato E, Funck-Brentano C, et al. 2019 ESC Guidelines for the diagnosis and management of chronic coronary syndromes. Eur Heart J. 2020 Jan 14;41(3):407–77.

2. Kunadian V, Chieffo A, Camici PG, Berry C, Escaned J, Maas AHEM, et al. An EAPCI Expert Consensus Document on Ischaemia with Non-Obstructive Coronary Arteries in Collaboration with European Society of Cardiology Working Group on Coronary Pathophysiology & Microcirculation Endorsed by Coronary Vasomotor Disorders International. European Heart Journal. 2020;41(37):3504–20.

3. Candell-Riera J, Martin-Comín J, Escaned J, Peteiro J. Physiologic evaluation of coronary circulation. Role of invasive and non invasive techniques. Revista Espanola de Cardiologia. 2002;55(3):271–91.

4. Rahman H, Corcoran D, Aetesam-Ur-Rahman M, Hoole SP, Berry C, Perera D. Diagnosis of patients with angina and non-obstructive coronary disease in the catheter laboratory. Heart. 2019 Oct 1;105(20):1536–42.

5. Ford TJ, Stanley B, Good R, Rocchiccioli P, McEntegart M, Watkins S, et al. Stratified Medical Therapy Using Invasive Coronary Function Testing in Angina: The CorMicA Trial. J Am Coll Cardiol. 2018 Dec 11;72(23 Pt A):2841–55.

6. A H. Functions of microRNAs in cardiovascular biology and disease. Annu Rev Physiol. 2013 Feb 10;75:69–93.

7. Widmer RJ, Chung WY, Herrmann J, Jordan KL, Lerman LO, Lerman A. The association between circulating microRNA levels and coronary endothelial function. PLoS One [Internet]. 2014 Oct 13 [cited 2022 Jan 28];9(10). Available from: https://pubmed.ncbi.nlm.nih.gov/25310838/

8. Rubio R, Berne RM. Regulation of coronary blood flow. Progress in Cardiovascular Diseases. 1975 Sep 1;18(2):105–22.

9. Hirshfeld JW, Nathan AS. Deriving Function From Structure: Applying Hagen-Poiseuille to Coronary Arteries. JACC: Cardiovascular Interventions. 2020 Feb 24;13(4):498–501.

10. De Bruyne B, Bartunek J, Sys SU, Heyndrickx GR. Relation between myocardial fractional flow reserve calculated from coronary pressure measurements and exercise-induced myocardial ischemia. Circulation. 1995 Jul 1;92(1):39–46.

11. Pijls NHJ, De Bruyne B, Simith L, Aarnoudse W, Barbato E, Bartunek J, et al. Coronary thermodilution to assess flow reserve: validation in humans. Circulation. 2002 May 28;105(21):2482–6.

12. Fearon WF, Balsam LB, Farouque HMO, Robbins RC, Fitzgerald PJ, Yock PG, et al. Novel index for invasively assessing the coronary microcirculation. Circulation. 2003 Jul 1;107(25):3129–32.

13. Pijls NHJ, Van Son JAM, Kirkeeide RL, De Bruyne B, Gould KL. Experimental basis of determining maximum coronary, myocardial, and collateral blood flow by pressure measurements for assessing functional stenosis severity before and after percutaneous transluminal coronary angioplasty. Circulation. 1993;87(4):1354–67.

14. Pijls NHJ, De Bruyne B, Peels K, Van Der Voort PH, Bonnier HJRM, Bartunek J, et al. Measurement of fractional flow reserve to assess the functional severity of coronary-artery stenoses. N Engl J Med. 1996 Jun 27;334(26):1703–8.

15. Svanerud J, Ahn JM, Jeremias A, Van ’T Veer M, Gore A, Maehara A, et al. Validation of a novel non-hyperaemic index of coronary artery stenosis severity: The Resting Full-cycle Ratio (VALIDATE RFR) study. EuroIntervention. 2018 Sep 1;14(7):806–14.

16. Scarsini R, Shanmuganathan M, de Maria GL, Borlotti A, Kotronias RA, Burrage MK, et al. Coronary Microvascular Dysfunction Assessed by Pressure Wire and CMR After STEMI Predicts Long-Term Outcomes. JACC Cardiovascular Imaging [Internet]. 2021 Apr 14 [cited 2022 Mar 3];14(10):1948–59. Available from: https://europepmc.org/article/MED/33865789

17. Çakmak HA, Demir M. Microrna and cardiovascular diseases. Balkan Medical Journal. 2020;37(2):60–71.

18. Lee RC, Feinbaum RL, Ambros V. The C. elegans heterochronic gene lin-4 encodes small RNAs with antisense complementarity to lin-14. Cell [Internet]. 1993 Dec 3 [cited 2022 Jan 24];75(5):843–54. Available from: https://pubmed.ncbi.nlm.nih.gov/8252621/

19. Lagos-Quintana M, Rauhut R, Lendeckel W, Tuschl T. Identification of novel genes coding for small expressed RNAs. Science [Internet]. 2001 Oct 26 [cited 2022 Jan 24];294(5543):853–8. Available from: https://pubmed.ncbi.nlm.nih.gov/11679670/

20. Lau NC, Lim LP, Weinstein EG, Bartel DP. An abundant class of tiny RNAs with probable regulatory roles in Caenorhabditis elegans. Science [Internet]. 2001 Oct 26 [cited 2022 Jan 24];294(5543):858–62. Available from: https://pubmed.ncbi.nlm.nih.gov/11679671/

21. Lee RC, Ambros V. An extensive class of small RNAs in Caenorhabditis elegans. Science [Internet]. 2001 Oct 26 [cited 2022 Jan 24];294(5543):862–4. Available from: https://pubmed.ncbi.nlm.nih.gov/11679672/

22. Wojciechowska A, Braniewska A, Kozar-Kamińska K. MicroRNA in cardiovascular biology and disease. Advances in Clinical and Experimental Medicine. 2017;26(5):865–74.

23. Hergenreider E, Heydt S, Tréguer K, Boettger T, Horrevoets AJG, Zeiher AM, et al. Atheroprotective communication between endothelial cells and smooth muscle cells through miRNAs. Nature Cell Biology 2012 14:3 [Internet]. 2012 Feb 12 [cited 2022 Apr 8];14(3):249–56. Available from: https://www.nature.com/articles/ncb2441

24. Oerlemans MIFJ, Mosterd A, Dekker MS, de Vrey EA, van Mil A, Pasterkamp G, et al. Early assessment of acute coronary syndromes in the emergency department: the potential diagnostic value of circulating microRNAs. EMBO Mol Med [Internet]. 2012 Nov [cited 2022 Apr 8];4(11):1176–85. Available from: https://pubmed.ncbi.nlm.nih.gov/23023917/

25. de Gonzalo-Calvo D, Dávalos A, Montero A, García-González Á, Tyshkovska I, González-Medina A, et al. Circulating inflammatory miRNA signature in response to different doses of aerobic exercise. J Appl Physiol (1985) [Internet]. 2015 Jul 15 [cited 2022 Mar 5];119(2):124–34. Available from: https://pubmed.ncbi.nlm.nih.gov/25997943/

26. Pijls NHJ, de Bruyne B, Smith L, Aarnoudse W, Barbato E, Bartunek J, et al. Coronary Thermodilution to Assess Flow Reserve. Circulation. 2002 May 28;105(21).

27. Calderon-Dominguez M, Belmonte T, Quezada-Feijoo M, Ramos-Sánchez M, Fernández-Armenta J, Pérez-Navarro A, et al. Emerging role of microRNAs in dilated cardiomyopathy: evidence regarding etiology. Translational Research [Internet]. 2020 Jan 1 [cited 2022 Apr 8];215:86–101. Available from: http://www.translationalres.com/article/S1931524419301707/fulltext

28. Harris TA, Yamakuchi M, Ferlito M, Mendell JT, Lowenstein CJ. MicroRNA-126 regulates endothelial expression of vascular cell adhesion molecule 1. Proc Natl Acad Sci U S A. 2008 Feb 5;105(5):1516.

29. Cengiz M, Yavuzer S, Avcı BK, Yürüyen M, Yavuzer H, Dikici SA, et al. Circulating miR-21 and eNOS in subclinical atherosclerosis in patients with hypertension. https://doi.org/103109/1064196320151036064. 2015 Nov 17;37(8):643–9.

30. Faraoni I, Antonetti FR, Cardone J, Bonmassar E. miR-155 gene: A typical multifunctional microRNA. Biochimica et Biophysica Acta (BBA) - Molecular Basis of Disease. 2009 Jun 1;1792(6):497–505.

31. Cordes KR, Sheehy NT, White M, Berry E, Morton SU, Muth AN, et al. miR-145 and miR-143 Regulate Smooth Muscle Cell Fate Decisions. Nature. 2009 Aug 6;460(7256):705.

32. Torella D, Laconetti C, Catalucci D, Ellison G, Leone A, Waring C, et al. MicroRNA-133 controls vascular smooth muscle cell phenotypic switch in vitro and vascular remodeling in vivo. Circ Res. 2011 Sep 30;109(8):880–93.

33. Liu Y, Song J-W, Lin J-Y, Miao R, Zhong J-C. Roles of MicroRNA-122 in Cardiovascular Fibrosis and Related Diseases. Cardiovascular Toxicology. 2020 Oct 1;20(5):1.

34. Pankratz F, Hohnloser C, Bemtgen X, Jaenich C, Kreuzaler S, Hoefer I, et al. MicroRNA-100 Suppresses Chronic Vascular Inflammation by Stimulation of Endothelial Autophagy. Circulation Research. 2018 Feb 2;122(3):417–32.

35. S DR, F E, C C, A S, G A, J S, et al. Transcoronary concentration gradients of circulating microRNAs in heart failure. Eur J Heart Fail. 2018 Jun 1;20(6):1000–10.

36. Eitel I, Adams V, Dieterich P, Fuernau G, De Waha S, Desch S, et al. Relation of circulating MicroRNA-133a concentrations with myocardial damage and clinical prognosis in ST-elevation myocardial infarction. American Heart Journal. 2012 Nov 1;164(5):706–14.

37. Fichtlscherer S, De Rosa S, Fox H, Schwietz T, Fischer A, Liebetrau C, et al. Circulating microRNAs in patients with coronary artery disease. Circ Res. 2010 Sep 3;107(5):677–84.

38. Zampetaki A, Willeit P, Tilling L, Drozdov I, Prokopi M, Renard JM, et al. Prospective Study on Circulating MicroRNAs and Risk of Myocardial Infarction. J Am Coll Cardiol. 2012 Jul 24;60(4):290–9.

39. Wang G-K, Zhu J-Q, Zhang J-T, Li Q, Li Y, He J, et al. Circulating microRNA: a novel potential biomarker for early diagnosis of acute myocardial infarction in humans. European Heart Journal. 2010 Mar 1;31(6):659–66.

40. Leistner DM, Boeckel J-N, Reis SM, Thome CE, De Rosa R, Keller T, et al. Transcoronary gradients of vascular miRNAs and coronary atherosclerotic plaque characteristics. European Heart Journal. 2016 Jun 7;37(22):1738–49.

41. J L, X D, Z W, J W. MicroRNA-1 in Cardiac Diseases and Cancers. The Korean Journal of Physiology & Pharmacology : Official Journal of the Korean Physiological Society and the Korean Society of Pharmacology. 2014 Oct 17;18(5):359–63.

42. Deng F, Xu X, Chen Y-H. The Role of miR-1 in the Heart: From Cardiac Morphogenesis to Physiological Function. Human Genetics & Embryology. 2014;4(1):1–4.

43. Jin Y, Yang C-J, Xu X, Cao J-N, Feng Q-T, Yang J. MiR-214 regulates the pathogenesis of patients with coronary artery disease by targeting VEGF. Molecular and Cellular Biochemistry 2015 402:1. 2015 Jan 10;402(1):111–22.

44. Chistiakov DA, Orekhov AN, Bobryshev Y V. Cardiac-specific miRNA in cardiogenesis, heart function, and cardiac pathology (with focus on myocardial infarction). Journal of Molecular and Cellular Cardiology. 2016 May 1;94:107–21.

45. Espinoza-Lewis RA, Wang DZ. MicroRNAs in Heart Development. Current Topics in Developmental Biology. 2012 Jan 1;100:279–317.

46. Tang Q, Lei H, Wu H, Chen J, Deng C, Sheng W, et al. Plasma miR-142 predicts major adverse cardiovascular events as an intermediate biomarker of dual antiplatelet therapy. Acta Pharmacologica Sinica. 2019 Feb 1;40(2):208.

47. Kaur A, Mackin ST, Schlosser K, Wong FL, Elharram M, Delles C, et al. Systematic review of microRNA biomarkers in acute coronary syndrome and stable coronary artery disease. Cardiovascular Research. 2021;116(6):1113–24.

48. de Gonzalo-Calvo D, Vilades D, Martínez-Camblor P, Vea À, Nasarre L, Sanchez Vega J, et al. Circulating microRNAs in suspected stable coronary artery disease: A coronary computed tomography angiography study. J Intern Med [Internet]. 2019 [cited 2022 Apr 8];286(3):341–55. Available from: https://pubmed.ncbi.nlm.nih.gov/31141242/

49. de Gonzalo-Calvo D, Martínez-Camblor P, Bär C, Duarte K, Girerd N, Fellström B, et al. Improved cardiovascular risk prediction in patients with end-stage renal disease on hemodialysis using machine learning modeling and circulating microribonucleic acids. Theranostics [Internet]. 2020 [cited 2022 Apr 8];10(19):8665–76. Available from: https://pubmed.ncbi.nlm.nih.gov/32754270/

50. Vilades D, Martínez-Camblor P, Ferrero-Gregori A, Bär C, Lu D, Xiao K, et al. Plasma circular RNA hsa_circ_0001445 and coronary artery disease: Performance as a biomarker. FASEB J [Internet]. 2020 Mar 1 [cited 2022 Mar 5];34(3):4403–14. Available from: https://pubmed.ncbi.nlm.nih.gov/31999007/

51. de Gonzalo-Calvo D, Vilades D, Martínez-Camblor P, Vea À, Nasarre L, Sanchez Vega J, et al. Circulating microRNAs in suspected stable coronary artery disease: A coronary computed tomography angiography study. J Intern Med [Internet]. 2019 [cited 2022 Mar 5];286(3):341–55. Available from: https://pubmed.ncbi.nlm.nih.gov/31141242/

52. de Gonzalo-Calvo D, Vilades D, Martínez-Camblor P, Vea À, Ferrero-Gregori A, Nasarre L, et al. Plasma microRNA Profiling Reveals Novel Biomarkers of Epicardial Adipose Tissue: A Multidetector Computed Tomography Study. J Clin Med [Internet]. 2019 Jun 1 [cited 2022 Mar 5];8(6). Available from: https://pubmed.ncbi.nlm.nih.gov/31159404/

53. Bustin SA, Benes V, Garson JA, Hellemans J, Huggett J, Kubista M, et al. The MIQE guidelines: minimum information for publication of quantitative real-time PCR experiments. Clin Chem [Internet]. 2009 Apr 1 [cited 2022 Mar 5];55(4):611–22. Available from: https://pubmed.ncbi.nlm.nih.gov/19246619/

54. Mestdagh P, Hartmann N, Baeriswyl L, Andreasen D, Bernard N, Chen C, et al. Evaluation of quantitative miRNA expression platforms in the microRNA quality control (miRQC) study. Nat Methods [Internet]. 2014 [cited 2022 Mar 5];11(8):809–15. Available from: https://pubmed.ncbi.nlm.nih.gov/24973947/

55. Sitia S, Tomasoni L, Atzeni F, Ambrosio G, Cordiano C, Catapano A, et al. From endothelial dysfunction to atherosclerosis. Autoimmunity Reviews. 2010 Oct 1;9(12):830–4.

56. Severino P, D’Amato A, Pucci M, Infusino F, Adamo F, Birtolo LI, et al. Ischemic Heart Disease Pathophysiology Paradigms Overview: From Plaque Activation to Microvascular Dysfunction. International Journal of Molecular Sciences. 2020 Nov 1;21(21):1–30.

57. Blanco-Domínguez R, Sánchez-Díaz R, Fuente H de la, Jiménez-Borreguero LJ, Matesanz-Marín A, Relaño M, et al. A Novel Circulating MicroRNA for the Detection of Acute Myocarditis. https://doi.org/101056/NEJMoa2003608. 2021 May 26;384(21):2014–27.

58. Casanova-Sandoval J, Fernández-Rodríguez D, Otaegu I, Jiménez TG, Rodríguez-Esteban M, Rivera K, et al. Usefulness of the Hybrid RFR-FFR Approach: Results of a Prospective and Multicenter Analysis of Diagnostic Agreement between RFR and FFR-The RECOPA (REsting Full-Cycle Ratio Comparation versus Fractional Flow Reserve (A Prospective Validation)) Study. J Interv Cardiol [Internet]. 2021 [cited 2022 Mar 3];2021. Available from: https://pubmed.ncbi.nlm.nih.gov/34007248/

59. Uribarri A, Nunez-Gil IJ, Conty DA, Vedia O, Almendro-Delia M, Cambra AD, et al. Short- and Long-Term Prognosis of Patients With Takotsubo Syndrome Based on Different Triggers: Importance of the Physical Nature. J Am Heart Assoc [Internet]. 2019 Dec 17 [cited 2022 Mar 3];8(24). Available from: https://pubmed.ncbi.nlm.nih.gov/31830875/

60. Gutiérrez E, Gómez-Lara J, Escaned J, Cruz I, Ojeda S, Romaguera y R, et al. <i class="fa fa-video-camera" aria-hidden="true"></i> Valoraci�n de la funci�n endotelial y provocaci�n de vasoespasmo coronario mediante infusi�n intracoronaria de acetilcolina. Documento t�cnico de la ACI-SEC. REC: interventional cardiology. 2021 Nov 16;

**Anexo 1: Consentimiento Informado.**

**HOJA DE INFORMACIÓN PARA PACIENTES**

**Estudio “****Caracterización epigenética de la angina pectoris según el compartimiento coronario afectado: relación entre la evaluación coronaria fisiológica invasiva y los microRNAs”.**

Por favor, lea atentamente la información facilitada en este documento, pregunte a su médico cualquier duda, y solicítele cualquier aclaración que considere necesaria, para que pueda decidir de forma libre y con la información necesaria si quiere o no participar en esta investigación.

**Introducción y objetivos:**

Se solicita su participación en este proyecto de investigación cuyo objetivo es determinar la asociación de la expresión de los miRNAs en función del compartimiento coronario afectado; en pacientes que sufren angina pectoris y se someten a un cateterismo cardíaco.

Las lesiones en las arterias del corazón (lesiones coronarias) pueden limitar la cantidad de sangre que llega al músculo cardiaco y producir síntomas como dolor de pecho (angina pectoris), dificultad respiratoria, y finalmente infarto de miocardio. Se sabe que la enfermedad de las arterias del corazón puede estar presente en las arterias principales (epicárdicas) o en las arterias microscópicas que nacen de las arterias principales (microcirculación). La valoración completa de esta enfermedad se realiza mediante un cateterismo cardiaco, que es una prueba invasiva que requiere de una punción arterial y que puede presentar, aunque no de forma frecuente, potenciales complicaciones.

Los microRNAs son unas moléculas que regulan determinados procesos fisiológicos, siendo liberadas a la sangre por las células y cuya concentración en sangre varía por diversas causas, siendo la enfermedad de las arterias del corazón una de ellas. Actualmente, el estudio preciso de la enfermedad de las arterias del corazón requiere de la realización de un cateterismo cardiaco, que como se menciona anteriormente, se trata de un procedimiento invasivo.

Los microRNAs modifican su concentración en sangre a causa de las lesiones en las arterias del corazón; y son una potencial herramienta para diagnosticar esta enfermedad de una forma mucho más sencilla, rápida segura y cómoda para el paciente, ya que para realizar su estudio solo se requiere una extracción sanguínea. Sin embargo, ésta es una técnica novedosa y todavía no se conoce de manera exacta cuáles son los microRNAs que modifican su concentración en sangre en pacientes con lesiones coronarias.

Este estudio pretende determinar cuáles son los microRNAs que modifican su concentración en pacientes con lesiones coronarias, así como determinar si existen patrones concretos que permitan distinguir si existe enfermedad en las arterias epicárdicas o en la microcirculación; para que esta técnica se pueda estandarizar y usar en el futuro de forma cotidiana en la práctica clínica habitual.

Este estudio está siendo evaluado por el Comité Ético de Investigación Médica (CEIM) del Hospital Universitario Arnau de Vilanova de Lleida y cumple los requisitos de la Ley de Investigación Biomédica 14/2007.

**Riesgos:**

La participación en el estudio no le implicará ningún inconveniente a nivel personal, ya que los estudios a realizar serán exactamente los mismos, tanto si acepta la participación en el estudio como si decide no participar. La única diferencia que existiría en caso de aceptar participar en el estudio sería que se realizaría una extracción sanguínea (sin requerir una punción adicional) que sería analizada y cuyos resultados junto con datos anonimizados, serían incluidos en una base de datos, para poder realizar el estudio.

**Beneficios:**

Usted no obtendrá ningún beneficio de su participación en este estudio. Sin embargo, la investigación de esta entidad podría tener implicaciones diagnósticas para los pacientes que estén en riesgo o la desarrollen en el futuro.

**Protección de datos personales:**

Se garantiza la confidencialidad y protección de los datos obtenidos de acuerdo con la legislación 2016/679 del Parlamento Europeo y del consejo del 27 de abril de 2016 Protección de Datos (RGPD); y de acuerdo con la Ley Orgánica 3/2018, de 5 de diciembre, de Protección de Datos Personales y Garantía de los Derechos Digitales (LOPD). De acuerdo con la ley vigente, tiene usted derecho al acceso de sus datos personales; asimismo, y si está justificado, tiene derecho a su rectificación y cancelación. Si así lo desea, deberá solicitarlo al Delegado de Protección de datos mediante el correo electrónico [dpd@ticsalutsocial.cat](mailto:dpd@ticsalutsocial.cat).

Mediante la firma del presente documento, usted consiente expresamente que tanto el centro como el investigador sean responsables respectivamente del tratamiento de sus datos y se comprometan a cumplir con la normativa de protección de datos en vigor.

El acceso a la información quedará restringido al personal que realiza la investigación, estando obligado a mantener la confidencialidad de los datos. Los resultados de la investigación podrán ser comunicados a las autoridades sanitarias y a la comunidad científica a través de congresos y publicaciones.

**Actividades del estudio:**

- Su médico realizará una recogida de datos de su historia clínica en el momento de realizar el cateterismo cardíaco.
- Extracción sanguínea durante el cateterismo cardíaco sin requerir de una punción adicional.
- No tendrá que venir a ninguna visita adicional.
- No se realizará ninguna actuación diferente a la práctica clínica habitual.

**Muestras biológicas e información asociada: en ningún caso se le practicará ninguna prueba experimental.**

Durante una de las extracciones de sangre que se le practicarán durante el proceso asistencial se recogerán 10 ml adicionales para realizar los estudios de investigación biomédica enmarcados dentro del presente proyecto de investigación. Este hecho no le causará molestias adicionales. Las muestras y la información asociada a las mismas se custodiarán en el hospital donde es atendido, bajo las condiciones y garantías de calidad y seguridad que exige la legislación vigente. Los análisis de las muestras se realizarán en los departamentos especializados de los hospitales implicados en este estudio.

La Dra. Lucía Matute Blanco, como coordinadora del programa, y el médico responsable de su tratamiento pondrán a su disposición toda la información relativa a los proyectos de investigación en los que se utilicen las muestras de sangre recogidas durante su participación en el estudio. El proceso se realiza siempre bajo la supervisión del hospital. La utilización de la muestra biológica para una finalidad distinta a la acordada en este documento tendrá que ser expresamente autorizada por usted en un nuevo documento de consentimiento.

**Carácter altruista de la donación. La cesión de muestras biológicas que usted realiza es gratuita.**

Usted no obtendrá ningún beneficio económico por su participación en los estudios de investigación, ni por un eventual beneficio derivado de los descubrimientos que pudieran realizarse en esta investigación biomédica.

**Participación voluntaria**

Su participación en el estudio es completamente voluntaria y usted puede negarse a participar sin ofrecer ninguna explicación. Así mismo se pone en su conocimiento que será atendido de la misma manera y con el mismo rigor profesional independientemente de su participación en este estudio.

**Revocación del consentimiento: si usted decide firmar este consentimiento, podrá también cancelarlo libremente. Ello conllevará la destrucción de sus muestras.**

Si en un futuro usted quisiera anular su consentimiento, sus muestras biológicas serían destruidas y los datos asociados a las mismas serían retirados. Sin embargo, los efectos de esta cancelación no se podrían extender retrospectivamente a la investigación que ya se hubiera llevado a cabo. Los derechos de acceso, rectificación, cancelación y oposición puede ejercitarlos ante:

Coordinador del proyecto: Lucía Matute Blanco

Hospital Universitario Arnau de Vilanova.

Dirección postal: Avenida Rovira Roure 80, CP 25198, Lleida, España.

Teléfono de contacto: 973-468024.

**Destino de las muestras tras su utilización en este proyecto de investigación.**

Una vez finalizado el proyecto de investigación las muestras sobrantes serán eliminadas.

**Persona de contacto:**

Durante la duración de esta investigación podrá formular cualquier pregunta que tenga. Si tiene algún problema o más preguntas sobre la misma o sobre sus derechos como paciente, póngase en contacto con la persona indicada a continuación

Médico responsable:

Dr/a ……………………..…………………………..…………………………..……

Dirección: …………………..……………………..…………………………..……

Teléfono: …………………………………………..…………………………..……

**HOJA DE CONSENTIMIENTO INFORMADO**

Esta hoja es específica para participar en el proyecto titulado: **“Caracterización epigenética de la angina pectoris según el compartimiento coronario afectado: relación entre la evaluación coronaria fisiológica invasiva y los microRNAs”**

Nombre y apellidos del Paciente: …………………………………………………

Fecha de Nacimiento: …………………………………………………………….

D.N.I.: ……………………………………………………………………………..

Centro: …………………………………………………………………………….

Si ha comprendido la información que se le ha proporcionado, ha resuelto cualquier duda que pudiese tener y decide colaborar con el presente proyecto de investigación en los términos anteriormente explicados, por favor, lea y firme a continuación esta hoja:

El abajo firmante autoriza al investigador responsable a almacenar y utilizar científicamente tanto la información clínico-asistencial de su historial médico como el material biológico como las pruebas de imagen que se le han realizado o se le van a realizar, con la finalidad de llevar a cabo el presente proyecto de investigación.

Confirmo que:

1. Autorizo que el excedente de material biológico utilizado para pruebas diagnósticas y la información clínica asociada se utilice para investigación asociada al proyecto de investigación arriba indicado: SI NO

2. Deseo que se me comunique la información derivada de la investigación que realmente sea relevante y aplicable para mi salud: SI NO

3. Autorizo a ser contactado en el caso de necesitar más información o muestras biológicas adicionales: SI NO

Además, estoy de acuerdo con los siguientes puntos:

1. He leído la hoja de información del estudio

2. He recibido la información necesaria

3. He hablado con el Dr/a …………………………………………………………….

y he tenido la posibilidad de preguntar acerca del proyecto.

4. Mi participación es voluntaria.

5. La participación no va a repercutir sobre la asistencia sanitaria que precise.

6. Todos los datos clínicos, resultados de pruebas y mi identidad no puede ser revelada sin mi permiso.

Firmado por (paciente): Firmado por (Investigador)

_____________________ _______________________

Fecha: Fecha:

**Anexo 2: Indicaciones generales para la realización de los estudios coronarios fisiológicos invasivos**(16,58,60)

| **En primer lugar, se realizarán las mediciones de RFR, RFRa, FFR, CFR e IMR que detallamos a continuación.** |
| --- |
| **Selección y Posicionamiento del Catéter**  • Usar catéteres guías (al menos 5F) sin agujeros laterales.  • Asegurar la canulación coaxial del catéter guía en el ostium coronario.  • Decanular el catéter guía del ostium para calibrar la presión, ecualizar y registrar el pd / pa si existen dudas acerca de que el catéter pueda obstruir parcialmente el ostium. |
| **Calibración**  • Antes de iniciar las mediciones, asegurarse de que la presión aórtica se ajusta a cero correctamente (1/3 versus 2/3 de diámetro del tórax).  • Purgar la guía de presión, colocarla en posición horizontal en el momento de conectar/calibrar y no mover la guía de presión mientras se realiza el proceso.  • Antes de ecualizar las presiones, avanzar la guía hasta que el sensor de presión se coloque exactamente en el extremo del catéter guía.  • Antes de igualar las presiones, lavar el catéter guía con suero fisiológico para eliminar el agente de contraste viscoso.  • Antes de igualar las presiones, retirar el introductor y cerrar la válvula hemostática.  • Las curvas de presión generalmente se promedian en tres a cinco latidos cardíacos. Por lo tanto, la ecualización de presión requiere algo de tiempo, y no deben producirse artefactos durante ese tiempo. |
| **Posicionamiento de la Guía de Presión-Temperatura**  • Para la evaluación de lesiones epicárdicas (FFR, RFR y RFRa), el sensor de presión debe colocarse en la distalidad del vaso principal a analizar.  • En primer lugar se realizará la medición de RFR y RFRa y posteriormente la medición de FFR, CFR e IMR.  • Para la determinación de CFR e IMR, el sensor de presión debe colocarse en el segmento distal del vaso correspondiente a la lesión a evaluar.  • En caso de pacientes remitidos para coronariografía que presenten test de isquemia positivo, también se realizará la determinación de FFR, CFR e IMR, además de en las arterias de las lesiones a evaluar, en las arterias compatibles con la isquemia detectada en dichos test de isquemia. Asimismo, en caso de ausencia de lesiones tributarias de evaluación y de test de isquemia positivo previo para orientar la exploración, el FFR, el CFR y el IMR se evaluarán en la descendente anterior.  • Una segunda guía, además de la guía de presión, podría provocar artefactos y, por lo tanto, debe evitarse.  • Detectar artefactos: el sensor podría interactuar con la pared del vaso, especialmente en casos de calibre de vaso estrecho o tortuosidad severa.  • El agente de contraste viscoso en la arteria coronaria puede afectar el gradiente pd/pa. |
| **Hiperemia**  • Antes del avanzar la guía de presión, administrar nitroglicerina ic (habitualmente 200 mcg) para prevenir el espasmo coronario.  - *Medicamentos para la hiperemia.*  - Adenosina endovenosa 140 μg / kg / min  - En caso de resultados de mediciones en el área límite, es posible un aumento de la dosis de adenosina ev. Sin embargo, dosis intravenosas> 180 μg / kg / min pueden reducir la perfusión coronaria y, por lo tanto, no se recomiendan. |
| **Registro y Evaluación**  • Los artefactos deben ser cuidadosamente observados y excluidos.  - *FFR, RFR y RFRa*  • Para la determinación del RFR, se requieren un mínimo de 5 ciclos cardiacos consecutivos.  • Tras la administración de adenosina intravenosa, los valores de presión pueden disminuir al mínimo antes de alcanzar un estado estable. Esperar hasta obtener valores estables.  • Los valores medidos durante bloqueo auriculoventricular / bradicardia inducido por la adenosina deben clasificarse como "no evaluables".  • Los latidos ectópicos pueden falsear los valores obtenidos, por lo que hay que asegurarse de obtener valores en periodos de tiempo de estabilidad eléctrica.  • Los valores del RFRa se obtienen a posteriori tras la transformación matemática mediante el ajuste del RFR por los factores de corrección.  - *CFR e IMR*  • Para la determinación del CFR y el IMR se realizarán 3 rápidas inyecciones de 3cc de suero salino fisiológico en situación basal. Posteriormente, durante la administración de adenosina ev y una vez se alcance el estado de máxima hiperemia se realizarán nuevamente 3 rápidas inyecciones de 3cc de suero salino fisiológico.  • Alteraciones en la temperatura del suero salino o de la velocidad de inyección del suero salino pueden condicionar valores anómalos de Tmn que son detectados por el software. Evaluar cuidadosamente la concordancia de dichos valores para valorar si hay que repetir alguna inyección.  • Para determinar el CFR se emplea la información del Tmn obtenido en situación basal y bajo hiperemia máxima. Para determinar el IMR solo se emplea la información del Tmn obtenido en hiperemia máxima. |
| **A continuación, se realizarán las mediciones de QFR e IMRangio con las especificaciones detalladas en el Software específico.** |
| **Únicamente se realizará Test de Acetilcolina para los pacientes de los Grupos 3 y 4 que detallamos a continuación.** |
| • Monitorización electrocardiográfica del paciente mediante electrocardiograma de 12 derivaciones.  • Las imágenes de cinefluoroscopia se obtendrán en una proyección craneal que despliegue adecuadamente la arteria descendente anterior.  • Dado el carácter previsiblemente difuso del espasmo coronario, se realizará mediante inyecciones crecientes de acetilcolina hasta 3 dosis de 2, 20 y 100 mcg en la coronaria izquierda.  • No se realizarán inyecciones de acetilcolina en la coronaria derecha dado el muy alto riesgo de bradicardia severa.  • El tiempo de administración del bolo intracoronario se realizará lentamente durante 20 segundos.  • Se debe lavar posteriormente de forma lenta el catéter guía con suero salino para prevenir inyecciones bruscas del fármaco remanente en el catéter en el momento de adquirir la imagen de cinefluoroscopia.  • Tras cada inyección se evaluará la presencia de síntomas sugestivos de angina y similares a los que motivaron el estudio, se adquirirá una imagen de cinefluoroscopia y se realizará un electrocardiograma de 12 derivaciones.  • En caso de documentarse espasmo angiográfico significativo o ascenso/descenso del ST sugestivo de espasmo micro o macrovascular no se continuará con la progresión a la siguiente dosis.  • En caso de espasmo significativo, así como al finalizar el test, se administrarán 200 mcg de NTG ic para abolir el efecto de la acetilcolina. |
